# Cost and effectiveness of differentiated ART service delivery strategies in Zambia: a modelling analysis using routine data

**DOI:** 10.1002/jia2.70003

**Published:** 2025-06-28

**Authors:** Nkgomeleng A. Lekodeba, Sydney Rosen, Bevis Phiri, Sithabiso D. Masuku, Caroline Govathson, Aniset Kamanga, Prudence Haimbe, Hilda Shakwelele, Muya Mwansa, Priscilla Lumano‐Mulenga, Amy N. Huber, Sophie J. S. Pascoe, Lise Jamieson, Brooke E. Nichols

**Affiliations:** ^1^ Health Economics and Epidemiology Research Office Faculty of Health Sciences University of the Witwatersrand Johannesburg South Africa; ^2^ Department of Global Health Boston University School of Public Health Boston Massachusetts USA; ^3^ Clinton Health Access Initiative Lusaka Zambia; ^4^ SCHARR, Sheffield Centre for Health and Related Research Division of Population Health, School of Medicine and Population Health University of Sheffield Sheffield England; ^5^ Department of Global Health Amsterdam Institute for Global Health and Development, Amsterdam UMC, University of Amsterdam Amsterdam Netherlands; ^6^ Ministry of Health Lusaka Zambia; ^7^ South African Centre for Epidemiological Modelling and Analysis (SACEMA) Stellenbosch University Stellenbosch South Africa

**Keywords:** antiretroviral treatment, cost‐effectiveness, differentiated service delivery, HIV, mathematical modelling, Zambia

## Abstract

**Introduction:**

Differentiated service delivery (DSD) models for antiretroviral treatment (ART) have been scaled up in many settings in sub‐Saharan Africa to improve client‐centred care and increase service delivery efficiency. However, given the multitude of models of care currently available, identifying cost‐effective combinations of DSD models that maximize benefits and minimize costs remains critical for guiding their expansion.

**Methods:**

We developed an Excel‐based mathematical model using retrospective retention and viral suppression data from a national cohort of ART clients (≥15 years) in Zambia between January 2018 and March 2022 stratified by age, sex, setting (urban/rural) and model of ART delivery. Outcomes (viral suppression and retention in care), provider costs and costs to clients were estimated from the cohort and published data. The base case reflects the outcomes observed in 2022 for all DSD models for each population sub‐group. For different combinations of nine DSD models and over 1‐year time horizon from the provider perspective, we evaluated the incremental cost‐effectiveness ratio (ICER) per additional client virally suppressed compared to the 2022 base case. Deterministic sensitivity analyses were conducted on key input parameters.

**Results:**

Among 125 scenarios evaluated, six were on the cost‐effectiveness frontier: (1) 6‐month dispensing (6MMD)‐only; (2) 6MMD and adherence groups (AGs); (3) AGs‐only; (4) fast track refills (FTRs) and AGs; (5) FTRs‐only; and 6) AGs and home ART delivery. 6MMD‐only was cost‐saving compared to the base case, increasing retention by 1.2% (95% CI: 0.7−1.8), viral suppression by 1.6% (95% CI: 1.0−2.7) and reducing client costs by 12.0% (95% CI: 10.8−12.4). The next cost‐effective scenarios, 6MMD + AGs and AGs‐only, cost $245 per additional person virally suppressed, increased viral suppression by 2.8% (95% CI: 2.2−3.3) and 4.0% (95% CI: 3.5−4.0) and increased client costs by 20.1% (95% CI: 9.5−28.1) and 52.3% (95% CI: 29.868.7), respectively. ART cost and laboratory test costs were the most influential parameters on provider costs and the ICERs.

**Conclusions:**

Mathematical modelling using existing data can identify cost‐effective DSD model mixes while ensuring all client sub‐populations are considered. In Zambia, scaling up 6MMD to all eligible clients is likely cost‐saving, with further health gains achievable by targeting sub‐populations with selected DSD models.

## 1 INTRODUCTION

Many countries in sub‐Saharan Africa are expanding differentiated service delivery (DSD) for HIV treatment to efficiently achieve national and global targets for treatment uptake and viral suppression [1, 2]. DSD models for HIV treatment differ from conventional care by location, interaction frequency, services and staff. They are designed to provide a more client‐centred approach, aiming to improve outcomes on antiretroviral treatment (ART) and reducing costs while improving experiences for clients and providers [1, 2]. Although it varies, conventional care is tailored to clients not eligible for DSD models, receiving < 3 months of medications and for those requiring more care.

The World Health Organization has recommended the implementation of DSD for HIV treatment since 2015 [3], and many countries have incorporated DSD models into national HIV treatment guidelines [4, 5, 6, 7]. Most of these models are “low intensity” approaches that require fewer resources from both clients and providers than conventional ART care [8]. These models include a 6‐month dispensing (6MMD) of ART medications, which reduces clinic visits to twice per year; community‐based medication pickup points; community clubs and groups; and fast‐track dispensing at facilities. Other models target specific population groups, such as teen/scholar clubs and extended clinic hours models for employed adults. Typically, each public sector HIV clinic offers some combination of the models recommended in its country's national guidelines, based on available resources and staff choices. Additionally, non‐governmental partner organizations often support several bespoke models at subsets of healthcare facilities, using donor funding.

As African countries continue to improve DSD to build the long‐term sustainability of their national HIV programmes, more attention is needed to identify optimal model mixes. Ideally, the allocation of models should reflect considerations of the trade‐offs and synergies among health outcomes, costs and benefits to clients, and costs and benefits to the healthcare system. While some evidence is available about each of these factors in some countries [2, 9, 10, 11, 12, 13], little is known about the cost‐effectiveness of different combinations of DSD models. In most settings, the distribution of models across facilities and nationally is largely determined by national guidelines, which are generally not tailored to local conditions, and by available facility and partner resources.

To assist in the prioritization and scale‐up of cost‐effective DSD models, we developed the Alternative Delivery of ART Optimization (ADAPT) model, an Excel‐based mathematical model that provides a decision‐making framework for scaling‐up DSD for ART, presented for the first time here. ADAPT compares different DSD model implementation scenarios in terms of viral suppression and providers’ and clients’ costs at a national level. We parameterized ADAPT for Zambia, a high HIV prevalence African country with extensive routine and research data, and then conducted a cost‐effectiveness analysis of likely combinations of ART delivery models to demonstrate how model mix and allocation may affect the achievement of HIV programme goals.

## 2 METHODS

We developed and parameterized an Excel‐based mathematical model of all ART clients in care in Zambia using de‐identified, retrospective SmartCare electronic data from Zambia's national cohort of ART clients (aged ≥15 years) from January 2018 to March 2022 [14]. SmartCare, Zambia's public sector electronic medical record system for HIV [11, 12, 13, 14, 15]. It includes data fields indicating when an ART client enrolled in a DSD model, the specific model enrolled in, the number of months for which ART medications were dispensed at each event, treatment outcome, outcome date and demographic characteristics [5, 16].

### 2.1 Study country

Zambia is a high HIV‐prevalence country with an estimated 1.3 million people living with HIV, and an estimated 95% of those living with HIV currently on ART [17]. The Zambian Ministry of Health supports a range of low‐intensity DSD models [8, 18]. Models described in national guidelines at the time of our data collection (2022) included 6MMD of ART at facilities, community medication pickup points, appointment spacing, fast‐track dispensing at facilities, teen/scholar clubs and adherence groups (AGs). At the time of our study, conventional care typically entailed a 3‐month dispensing of ART at health facilities, with a clinical consultation and medication pickup at each quarterly clinic visit. Not all models were offered by all facilities. Further description of the models in use is provided below.

### 2.2 Model structure and approach

To determine the expected impact and cost‐effectiveness of various combinations of DSD models, we started with a base case representing the actual 2022 national distribution of ART clients among DSD models disaggregated by population sub‐groups (age group, sex, urban or rural location). The health outcomes from this base case reflect the health outcomes observed in 2022 for all DSD models for each population sub‐group.

**Figure 1:**
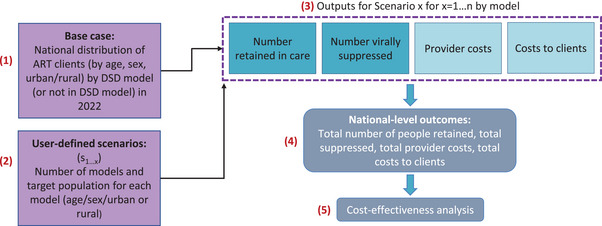
ADAPT modelling structure.

We then constructed multiple hypothetical scenarios in which specific combinations of one or more DSD models were implemented across the entire population cohort or be targeted to specific sub‐populations based on age, sex and urban or rural setting. Each scenario reflected a specific distribution of ART clients among different models. The model then generated a total number of individuals virally suppressed and retained in care (the sum of all individuals virally suppressed on treatment and retained in care by the model of ART delivery for each scenario), and a total estimated cost to the provider and clients. Figure 1 outlines the five‐step modelling approach.

### 2.3 Cohort, eligibility and outcomes

The primary outcome for this analysis was incremental costs to the provider per additional person suppressed on treatment over 1‐year time horizon. We also estimated retention in care, viral suppression among those retained in care, provider costs and cost to ART clients. Viral suppression and provider costs were combined into an incremental cost‐effectiveness ratio (ICER) for each scenario; costs to clients were not included in the estimation of the ICERs but were considered separately. Study cohort, eligibility criteria and outcomes are defined in Table 1.

**Table 1:** Definitions of study cohort, eligibility criteria and outcomes

Cohort, criterion or outcome	Definition
*Study cohort*	
Study analytic cohort	The study analytic cohort comprised ART clients (aged ≥15 years) included in the SmartCare database in January 2018−March 2022 and enrolled in differentiated or conventional care.
*Criteria*	
Eligibility for DSD enrolment	On ART for at least 12 months. (Because there were limited viral load data available, viral suppression could not be used as an eligibility criterion despite it being specified in DSD policy guidelines.)
DSD model assignment	Each participant was assigned to the first DSD model they enrolled in during the first year after becoming eligible for DSD. Those who did not have any DSD model enrolment reported in the dataset were classified as remaining in conventional care.
*Outcomes*	
Total provider costs	The sum of provider costs per year
Total costs to clients	The sum of cost to clients incurred accessing ART services per year
Retention in care at 12 months	The proportion of ART clients who had a clinic visit between 11 and 24 months (the wide range to account for the fact that facility interactions occur as little as once per year with some DSD models) stratified by age group, sex, setting (urban/rural) and model of ART delivery. Retention in care for clients enrolled in DSD model was estimated after they became eligible and were enrolled in DSD models.
Viral suppression at 12 months	The proportion of ART clients who had viral load test results of <1000 copies/ml at 12 months, stratified by age group, sex, setting (urban/rural) and model of ART delivery. The viral load at 12 months is measured between 11 and 24 months (the wide range to account for the fact that facility interactions occur as little as once per year with some DSD models).
Incremental cost‐effectiveness ratio (ICER)	Healthcare system (provider) cost per additional ART client virally suppressed on treatment

### 2.4 ART delivery models

In the modelled scenarios, we considered a total of 11 models of ART delivery for ART clients in 2022 (Table 2). Two were variations of conventional (non‐differentiated) care: 3‐month facility‐based dispensing (3MMD), which is the current standard of care, and a study‐defined model for clients observed to be receiving less than 3 months of medication at a time. Nine were DSD models [11, 19]. At the time of the study, Zambia's criteria for being designated as “established on treatment” were a viral load of <1000 copies/ml and a minimum of 6 months on ART. ART clients in conventional care went through all service points (registration, vitals, clinical consultation, pharmacy, etc.) within the facility at each quarterly visit.

**Table 2:** Description of conventional and differentiated ART service delivery models

Category	ART delivery model	Description
**In‐facility models**	Conventional care not eligible for DSD	Conventional care for ART clients who are shown in the database to receive <3‐month refills of medications. Reasons for shorter dispensing intervals are not indicated in the database but may include insufficient experience on ART, unsuppressed viral loads, missed medication refills, uncontrolled co‐morbidities; some clients may receive shorter refills at their own request or due to clinic stock limitations. ART clients receiving <3‐month refills go through all service points (registration, vitals, clinical consultation, pharmacy, etc.) within the facility at each visit. (Note: “Conventional care not eligible for DSD” was defined for this analysis to distinguish it from standard of care for clients who are eligible for DSD.)
	Conventional care (3MMD)	Conventional care targeted to ART clients not yet enrolled in other models, declining other models or not enrolled in DSD for some other reason.
	Fast‐track refills (FTR)	Established clients access a separate shorter queue for medication collection visits and spend less time during the service interactions with providers; medication refill duration or dispensing intervals varies between 3 and 4 months.
	Six‐month dispensing (6MMD)	Clients are dispensed 6 months of ART medication at each clinic visit. Clinic visits are scheduled every 6 months, with no required interactions with the healthcare system between visits.
	Extended clinic hour models	ART clients aged ≥25 years enrolled in these models of care are eligible to collect medication outside facility operation hours (e.g. morning, evening or weekends).
	Scholar/adolescent models	Group model for teenagers and school‐going population aged between 10 and 24 years. The groups meet four times a month to discuss treatment adherence and 3 months ART medications are dispensed every 3 months during the group meetings. (Note: For this analysis, we only included teenagers and school‐going population aged between 15 and 24 years.)
**Out‐of‐facility‐based models**	Adherence groups (rural and urban)	Up to 30 members who meet with a healthcare worker every 1–3 months at the facility to collect pre‐packed medications, share treatment experiences and reinforce medication adherence practices.
	Community ART distribution points	ART refills are provided at central location within the community such as in schools, churches, community halls, retail pharmacies and health posts.
	Health posts	ART medications are dispensed at the health post which is linked to a main healthcare facility. These are usually situated in remote areas where access to health facilities may be limited. ART clients receive 3–6 months of ART medication at each.
	Mobile ART delivery	A clinical outreach team who are linked to a facility conducts 3‐monthly clinical assessments in rural settings.
	Home ART delivery (HAD)	A trained healthcare worker visits ART client in their homes to conduct health screening, provide pre‐packed medications and monitor adherence. ART clients eligible are those ≥25 years.

The nine DSD models considered in the analysis were categorized as either in‐facility or out‐of‐facility models and are described in Table 2 [5]. We excluded models tailored to clients with viral loads >1000 copies/ml, such as “high viral load clinics.” Instead, cohort participants who did not meet DSD eligibility criteria were retained in conventional care in our mathematical model.

### 2.5 Input parameters and assumptions

Model input parameters, parameter values, client characteristics and the base case distribution of clients that reflect the 2022 cohort are presented in Table 3. Health outcomes (retention in care and suppression rates) for each ART delivery model, stratified by sex, age and setting, were estimated from the SmartCare database (Table 3, Text S1 and Table S1) [14].

**Table 3:** Cohort and outcomes input parameters (*N* = 917,749)

Parameter	Model value, *n* (%)
*Demographic characteristics* [14]	
Sex (female)	584,509 (63.7%)
Setting (urban)	636,597 (69.3%)
Age group	
15−19 years	21,608 (2.4%)
20−24 years	49,990 (5.4%)
25−49 years	660,085 (71.9%)
50+ years	186,066 (20.3%)

Distribution of ART delivery models (n, %) [14]	Male	Female
Conventional care not eligible for DSD	17,914 (5.4%)	30,766 (5.3%)
Conventional care (3MMD)	47,302 (14.2%)	85,728 (14.7%)
Six months dispensing	231,153 (69.4%)	401,085 (68.6%)
Fast‐track refills	29,096 (8.7%)	52,691 (9.0%)
Extended clinic hours	75 (<0.1%)	130 (<0.1%)
Scholar/adolescent model	92 (<0.1%)	110 (<0.1%)
Mobile ART distribution	583 (0.2%)	1159 (0.2%)
Health post	691 (0.2%)	1281 (0.2%)
Home ART delivery	1366 (0.4%)	2020 (0.3%)
Community ART distribution points	2693 (0.8%)	4989 (0.9%)
**Adherence groups**	2275 (0.7%)	4550 (0.8%)

*Average health outcomes* [14]	*Retention rates*	*Suppression rates*
Conventional care not eligible for DSD	77.7% (75.8; 79.5)	80.1% (77.3; 82.6)
Conventional care (3MMD)	86.4% (85.3; 87.3)	85.5% (83.8; 87.0)
Six months dispensing	90.6% (87.3; 94.2)	90.6% (86.7; 93.7)
Fast‐track refills	89.7% (82.5; 98.2)	95.5% (89.9; 97.7)
Extended clinic hours	90.2% (80.8; 94.6)	98.3% (84.2; 99.7)
Scholar/adolescent model	93.5% (74.6; 98.6)	87.5% (65.5; 95.6)
Mobile ART distribution	95.3% (89.6; 97.9)	89.8% (75.1; 96.3)
Health post	89.3% (83.5; 93.1)	95.5% (84.1; 98.3)
Home ART delivery	90.9% (86.7; 98.2)	95.0% (91.4; 97.9)
Community ART distribution points	92.0% (84.0; 95.2)	88.4% (76.2; 95.2)
Adherence groups	91.7% (82.8; 97.2)	95.9% (92.2; 97.9)

Provider costs using a micro‐costing approach, and costs to clients from sentinel sites across Zambia, were estimated using previously published primary cost data of DSD models in Zambia (Table 4) [20, 21]. These costs included visits to the healthcare facility, DSD interactions, ART medication, non‐ART medication and laboratory tests [20]. All costs are presented in 2023 USD. Additional detail on cost inputs can be found in Text S2 and Tables S2 and S3.

**Table 4:** Cost inputs—provider costs and cost to client per year

		Average costs to clients per year [21]		
			Transport costs	
Costs per year (2023 USD)	Average provider costs per client per year [20]	Lost wages (opportunity costs), (95% CI)	Incurring any costs (%)^a^	Average cost (95% CI)^a^
Conventional care not eligible for DSD	$119.23	$6.94 (5.60; 8.28)	51.5%	$3.19 (1.98; 4.40)
Conventional care (3MMD)	$109.05	$5.01 (4.33; 5.70)	40.9%	$3.01 (0.89; 5.14)
Six‐month dispensing	$99.72	$3.01 (2.50; 3.51)	36.4%	$2.80 (1.56; 4.04)
Fast‐track refills	$105.90	$3.94 (2.94; 4.95)	42.5%	$1.32 (0.86; 1.77)
Extended clinic hours	$109.05	$2.35 (1.94; 2.76)	46.7%	$1.74 (0.56; 2.91)
Scholar/adolescent model	$109.05	$4.34 (3.19; 5.48)	26.8%	$1.66 (0.64; 2.68)
Mobile ART distribution	$125.56	$4.01 (2.10; 5.92)	0.0%	$0.0 (0.0; 0.0)
Health post	$107.27	$5.21 (3.94; 6.49)	51.4%	$1.71 (1.04; 2.38)
Home ART delivery	$132.51	$2.67 (2.19; 3.16)	40.0%	$2.98 (1.16; 4.81)
Community ART distribution points	$106.19	$5.21 (3.94; 6.49)	51.4%	$1.71 (1.04; 2.38)
Adherence groups	$104.33	$6.44 (4.45; 8.42)	34.8%	$2.09 (0.14; 4.31)

^a^ Among clients incurring any transport costs, the remainder likely walked.

### 2.6 Scenarios and input assumptions

As noted above, the base case was defined as the current national distribution of ART clients by age, sex, setting and ART delivery model type, under the current status quo, as determined by the SmartCare database. The analytic scenarios include 125 unique possible combinations of DSD models and model offerings tailored by age, sex and setting. While most DSD models allow enrolment of any eligible ART patient, some are limited to specific population sub‐groups. These include the scholar/adolescent model (for ART clients aged ≤24 years), extended clinic hours (for clients aged ≥25 years) and mobile ART delivery (available only in rural areas). For scenarios with two or more DSD models, we assumed an equal distribution of the number of eligible ART clients allocated to each model. Further details on DSD model allocation can be found in Text S3 and Table S4.

### 2.7 Cost‐effectiveness analysis

We conducted an incremental cost‐effectiveness analysis from the provider perspective across all scenarios and eliminated those that were strongly dominated (higher costs and fewer people virally suppressed) or weakly dominated (lower effectiveness but higher ICERs compared to the next best scenario). ICERs were calculated for each scenario on the cost‐effectiveness frontier by dividing the incremental cost by the incremental number of people virally suppressed compared to the next best scenario. Scenarios on the cost‐effectiveness frontier were used to assess health outcomes (viral suppression and retention) by age, sex and rural/urban setting at a national level compared to the base case. Finally, for all interventions on the cost‐effectiveness frontier, a sub‐analysis was conducted to understand how health outcomes (viral suppression and retention rates) of each sub‐group (by age, sex and rural/urban setting) would be affected compared to base case distribution.

### 2.8 Sensitivity analysis

We conducted univariate and multivariate deterministic sensitivity analysis to examine the impact of differences in cost on the ICER per person suppressed (Table S2 and Text S4). To assess the impact of the uncertainty in effectiveness on the cost per person suppressed, we used the upper and lower bounds of the 95% confidence intervals (CIs) of the number suppressed of each model and sub‐population (Table S5) for the scenarios on the cost‐effectiveness frontier in the primary analysis.

### 2.9 Ethics statement

The study protocol for this analysis was approved by ERES Converge IRB (Zambia), protocol number 2019‐Sep‐030; the Human Research Ethics Committee (Medical) of the University of Witwatersrand (South Africa), protocol number M190453; and the Boston University IRB (United States), protocol number H‐38823. All three approved the use of routine clinic data and a waiver of consent.

## 3 RESULTS

### 3.1 Base case scenario

The base case cohort comprised a total of 917,749 ART clients, of whom roughly two‐thirds were female (63.7%) and two‐thirds live in an urban setting (69.3%) (Table 3). Most (72%) were aged between 25 and 49 years, and another quarter were ≥50 years, leaving just 8% ≤24 years. In the base case, reflecting the status quo in 2022, less than a quarter of the cohort (20%) received conventional care, nearly 70% 6MMD, 9% fast track, and the remainder were enrolled in the other active models, which served fewer than 1% of clients each. The average provider cost ranged from $100 to $133 per client per year, while costs to clients ranged from $2.35 (95% CI: 1.94; 2.76) to $6.94 (95% CI: 5.60; 8.28) per year for lost wages. Among the 39.2% of those who incur transport costs, the cost per client ranged from $1.32 (95% CI: 0.86; 1.77) to $3.19 (95% CI: 1.98; 4.40) per year (Table 5).

**Table 5:** Health outcomes and costs for the Zambia ART programme at 12 months under the base case, 2022

Characteristics	National outcomes, *n*, %	% (95% CI)
**Analytic cohort**		
**Total (*N*)**	917,749	100% (n/a)
Retained in care	817,948 (95% CI: 786,510−855,901)	89.1% (85.7−93.3)
Suppressed on treatment	770,086 (95% CI: 717,521−823,735)	94.1% (91.2−96.2)
**Costs (2023 USD)**		
Provider costs	$84,332,234 (n/a)	n/a
Costs to clients	$4,145,400 (95% CI: 3,071,187−4,951,395)	n/a
**DSD eligibility/enrolment** ^a^		
Conventional care not eligible for DSD	48,679 (5.3%)	n/a
Conventional care eligible for DSD but not enrolled	133,031 (14.5%)	n/a
Clients enrolled in DSD models	736,039 (80.2%)	n/a

^a^ Clients enrolled in DSD models and those eligible but not enrolled can be allocated to any DSD model for the scenario analysis; clients not eligible for DSD must remain in conventional care.

A total of 89.1% (95% CI: 85.7; 93.3) of the cohort were retained in care at 12 months in the base case scenario (Table 5). Among those retained in care, 94.1% (95% CI: 91.2; 96.2) were virally suppressed. Of the entire cohort, 80.2% (*n* = 736,039) were enrolled in DSD models, 14.5% (*n* = 133,031) were eligible for DSD but not enrolled and 5.3% (*n* = 48,679) were not eligible for DSD enrolment. The associated provider and client costs were $84,332,234 and $4,145,400 per year, respectively.

### 3.2 Scenarios on the cost‐effectiveness frontier

**Figure 2:**
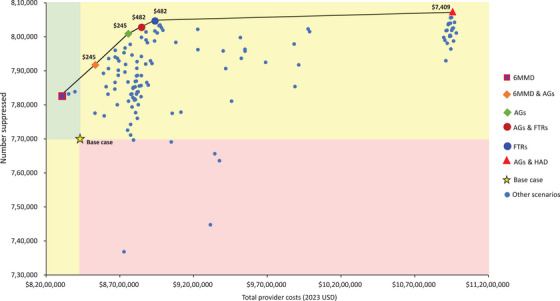
Total number of people suppressed on treatment by total provider costs for scenarios on the cost‐effectiveness frontier.

The results of all 125 scenarios analysed from the provider perspective are reported in Table S6. Six of these scenarios were found to be on the cost‐effectiveness frontier (Tables 6, 7, and Figure 2). Five of the scenarios on the frontier improved outcomes but also resulted in greater provider costs and costs to clients, while one scenario—6MMD for all eligible clients—both improved outcomes and reduced costs, compared to the base case. Scenarios on the cost‐effectiveness frontier encompassed both facility‐based models (6MMD, fast‐track refills) and out‐of‐facility‐based models (AGs, home ART delivery) for eligible clients. The only scenario on the cost‐effectiveness frontier that included an approach that targeted specific sub‐populations was the combination of AGs (for those ≤24 years) and home ART delivery (HAD) (for those aged ≥25).

**Table 6:** Description of DSD scenarios on the cost‐effectiveness frontier

Scenarios	Description
Base case	Current distribution of ART delivery models as indicated in the SmartCare database (80.2% enrolled in DSD, 14.5% enrolled in conventional care and eligible for DSD but not enrolled, and 5.3% in conventional care but not eligible for DSD).
6MMD	94.7% (*n* = 869,070) of all ART clients enrolled in 6MMD across all population groups (rural/urban, age, sex) (all of those eligible for DSD models), and 5.3% (*n* = 48,679) remains in conventional care (not eligible for DSD models).
6MMD and AGs	94.7% (*n* = 869,070) of all ART clients distributed equally between a combination of two DSD models (6MMD and AGs) and 5.3% (*n* = 48,679) remains in conventional care (not eligible for DSD models).
AGs	94.7% (*n* = 869,070) of all ART clients enrolled in AGs across all population groups (rural/urban, age, sex) and 5.3% (*n* = 48,679) remains in conventional care not eligible.
FTRs and AGs	94.7% (*n* = 869,070) of all ART clients distributed equally between a combination of two DSD models (FTRs and AGs) across all population groups (rural/urban, age, sex) and 5.3% (*n* = 48,679) remains in conventional care (not eligible for DSD models).
FTRs	94.7% (*n* = 869,070) of all eligible clients enrolled in FTRs only across all population groups (rural/urban, age, sex) and 5.3% (*n* = 48,679) remains in conventional care (not eligible for DSD models).
AGs and HAD	Population‐specific scenario for all 94.7% (*n* = 869,070) eligible clients; enrolling 6.8% (*n* = 62,106) of clients aged ≤24 years in AGs and 87.7% (*n* = 806,964) of clients aged ≥25 years in HAD across all population groups (rural/urban, age, sex) and 5.3% (*n* = 48,679) of all clients remain in conventional care (not eligible for DSD models).

Abbreviations: 6MMD, six‐month dispensing; AGs, adherence groups; FTRs, fast track refills; HAD, home ART delivery.

**Table 7:** Health outcomes, provider costs and ICERs for the DSD scenarios on the cost‐effectiveness frontier

Scenarios	Total number of people retained, *n*, 95% CI (% change compared to base case)	Total number of people suppressed, *n*, 95% CI (% change compared to base case)	Total provider cost, USD (% change compared to base case)	ICER per additional person suppressed (USD)^a^
Base case	817,948 (786,510; 855,901) (n/a)	770,086 (717,521−823,735) (n/a)	$84,332,234 (n/a)	n/a
6MMD	827,415 (792,037; 871,306) 1.2% (0.7; 1.8)	782,545 (724,788−846,146) 1.6% (1.0; 2.7)	$83 095,136 (−1.5%)	Cost‐saving (compared to base case)
6MMD and AGs	831,498 (797,906; 874,787) 1.7% (1.4; 2.2)	791,712 (733,632−851,998) 2.8% (2.2; 3.4)	$85,336,549 (1.2%)	$245
AGs	835,581 (803,774−878,268) 2.2% (2.2; 2.6)	800,878 (742,477−857,849) 4.0% (3.5; 4.1)	$87,577,961 (3.8%)	$245
FTRs and AGs	838,225 (802,976−886,582) 2.5% (2.1; 3.6)	802,755 (739,700−865,063) 4.2% (3.1−5.1)	$88,483,273 (4.9%)	$482
FTRs	840,869 (802,178−894,895) 2.8% (2.0; 4.6)	804,632 (736,924; 874,277) 4.5% (2.7−6.1)	$89,388,584 (6.0%)	$482
AGs and HAD	844,609 (800,901−890,409) 3.3% (3.0−4.0)	807,356 (745,765−871,801) 4.8% (3.9−5.8)	$109,574,215 (29.9%)	$7409

Abbreviations: 6MMD, six‐month dispensing; AGs, adherence groups; FTRs, fast track refills; HAD, home ART delivery.

^a^ ICERs per additional person suppressed on treatment were calculated by comparing scenarios to the previous scenarios that was on the cost‐effective frontier.

With the exception of 6MMD, which was cost‐saving, all other scenarios on the cost‐effectiveness frontier incurred additional costs per additional person virally suppressed, with ICERs ranging from $245 to $7409. Compared to the base case, 6MMD increased retention by 1.2% (95% CI: 0.7; 1.8) and viral suppression by 1.6% (95% CI: 1.0; 2.7), while reducing provider costs by 1.5%. As shown in Figure 2, the remaining scenarios improved outcomes further but increased costs. The most expensive scenario—AGs for young adults and HAD for others—achieved the largest gains in retention (3.3%; 95% CI: 3.0; 4.0) and suppression (4.8%; 95% CI: 3.9; 5.8) at a nearly 30% higher annual cost.

### 3.3 Costs to ART clients

**Figure 3:**
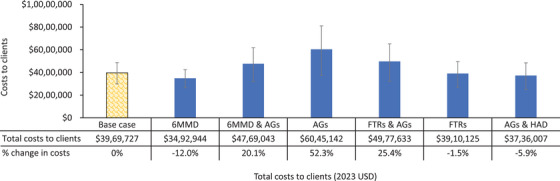
Cost to clients per year for the scenarios on the cost‐effectiveness frontier (2023 USD).

From a client perspective, compared to the current base case ART distribution, 6MMD‐only, FTRs‐only, and a combination of AGs and HAD were cost‐saving, while the other scenarios on the cost‐effectiveness frontier increased client costs (Figure 3 and Table S7). Enrolling all eligible clients in 6MMD‐only reduced client costs by 12.0% (95% CI: −10.8; −12.4), while combining 6MMD and AGs increased costs by 20.1% (95% CI: 9.5; 28.1). The highest cost scenario—AGs‐only—raised client costs by 52.3% (95% CI: 29.8; 68.7), while FTRs and AGs combined led to a 25.4% (95% CI: 11.6; 36.0) increase. In contrast, FTRs‐only and the age‐specific scenario (AGs ≤24 years, HAD ≥25 years) reduced client costs by 1.5% (95% CI: −7.3; +3.3) and 5.9% (95% CI: −15.6; +0.5), respectively. The sub‐population outcomes for each scenario on the cost‐effectiveness frontier are reported in Text S5 and Table S8.

### 3.4 Sensitivity analysis

**Figure 4:**
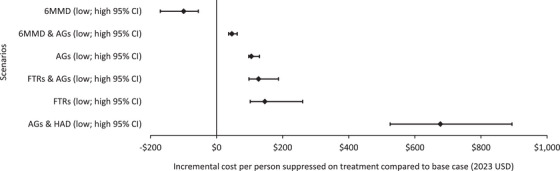
Sensitivity analysis of changes in model effectiveness (using low and high bounds from the 95% CI effectiveness ranges across all models).

Our primary conclusion that a switch to 6MMD‐only would result in improved outcomes and decreased costs was robust in our sensitivity analysis of surrounding model effectiveness (Figure 4). Variability in outcomes for 6MMD+AGs was also limited ($37−$62). There was significant overlap, however, in expected cost per person suppressed for AGs‐only, FTRs+AGs and FTRs only‐ ($98−$130, $98−$137 and $102−$261, respectively, compared to base case). This would suggest that when prioritizing between any of those scenarios, other outcomes should be considered in tandem (such as cost to the person seeking care) when choosing between model combinations. The greatest variability was in the AGs+HAD scenario ($525−$894 per person suppressed compared to the base case), likely driven by the uncertainty range around the effectiveness of HAD specifically. The impact of changes in cost assumptions can be found in Text S6 and Figures S1 and S2.

## 4 DISCUSSION

In this study, we identified DSD scenarios likely to improve health outcomes over the current ART distribution in Zambia and estimated their additional cost to the HIV programme budget. Enrolling all eligible clients in 6MMD was cost‐saving for both providers and clients while improving outcomes modestly. Given the current funding landscape for HIV treatment and care, scenarios that resulted in cost‐savings while improving outcomes should be prioritized. Other DSD combinations on the cost‐effectiveness frontier, though not cost‐saving, could increase retention and viral suppression by up to 4.8% (95% CI: 3.9; 5.8) but come at additional costs. Although these models are more expensive than the base case (with 70% of clients in 6MMD in the base case), they may reduce total provider costs in the long term by lowering transmission. Before opting for a combination of DSD models that results in a cost increase, these long‐term effects should be explicitly assessed.

The 6MMD‐only scenario improved retention and suppression for all sub‐groups except clients ≥50 years in urban settings. While 6MMD‐only improved health outcomes for other sub‐populations, further gains could be prioritized by national health programmes. For example, offering AGs or home delivery for young women could improve outcomes for these target groups, without significantly increasing resources. This suggests that tailored DSD models may be needed for specific sub‐groups, rather than a single scale‐up strategy for all eligible clients. The cost‐effectiveness frontier scenarios were most sensitive to ART drug and lab test costs, except for population‐specific scenarios (AGs for ≤24 years and HAD for clients ≥25), where DSD interaction costs had the greatest impact.

To our knowledge, this is the first model to explore how distributing DSD models might affect a national HIV treatment programme's cost‐effectiveness. Previous studies have examined the outcomes and costs of individual DSD models, with varying results depending on the country and model(s) evaluated [2, 10, 22], or compared the costs of DSD models to “no DSD” scenarios [13]. Across countries, retention and suppression rates in DSD models have generally been within 5% of conventional care [2]. A Zambian retrospective review showed that community DSD models were more expensive than conventional care, with annual provider costs ranging from $116 to $199, compared to $100 for conventional care [20]. A cluster‐randomized trial in Zambia and Malawi showed that 6‐month ART dispensing had non‐inferior retention and uptake compared to 3‐month dispensing, and was cost‐saving for both the healthcare system and clients [23]. Trials in Lesotho and Zimbabwe showed that community AGs with 3‐ and 6‐month medication dispensing were less costly than conventional care, with similar health outcomes [24, 25]. A Mozambique analysis concluded that DSD is less expensive than conventional care in a hypothetical scenario [13]. These findings support our conclusion that DSD models can improve health outcomes and minimize costs.

Although specific to Zambia, our study offers insights for policy‐makers considering how changes in DSD models may impact health outcomes, costs and prioritization of population‐specific model combinations. If cost‐savings are the priority without sacrificing outcomes, implementing 6MMD for all eligible clients could achieve this goal. However, if improving health outcomes while allowing for a modest budget increase is the goal, models with low ICERs may be optimal. The higher client costs associated with non‐conventional models, along with client preferences, must also be considered.

Our study's strengths include using a national dataset to quantify outcomes of different DSD models, local cost estimates and analysing multiple hypothetical model combinations. However, there are limitations. First, we parameterized the model using retention and viral suppression rates from individuals enrolled in models as of the end of 2022. Our results do not capture crossover effects on outcomes of ART clients transitioning from the current distribution of DSD models to the modelled scenarios, which may differ in health outcomes and costs, especially if the base case reflects client preferences. Thus, results should be seen as illustrative, not precise predictions.

Second, we only modelled national‐level outcomes with rural and urban stratification, without considering provincial or geographic differences. The results may vary in provinces with differing settings and poor access to care. Third, due to a lack of actual cost data for all care models, we used ingredient‐based costing. Actual costs may be over‐ or underestimated. Fourth, client costs were estimated from self‐reported visit frequencies, time spent, lost wages and transport costs, which may be biased due to recall errors. Fifth, we did not account for additional coordination and management costs required to implement multiple care models at a single site, nor did we capture higher‐level costs such as national or provincial planning, training and budgeting. Some facilities may struggle with implementing multiple models, requiring extra resources. However, none of the cost‐effectiveness frontier scenarios required more than two non‐conventional models, minimizing concerns about coordination burden.

Sixth, our findings may not be generalizable beyond Zambia, as the cost‐effectiveness of DSD models is context dependent. The results may be applicable to countries in southern Africa with similar conditions. Seventh, we included a liberal range to define retained in care and virally suppressed due to the limitations of the data. It could be that there were periods of non‐retention or viral non‐suppression that occurred during these intervals. We do not expect, however, that these periods of non‐suppression would vary by model of care. Finally, sensitivity analyses were conducted only for scenarios on the cost‐effectiveness frontier. Given the uncertainty around viral suppression rates for sub‐populations, it is likely that any scenario close to the cost‐effectiveness frontier may also represent a cost‐effective combination of models of care. Decisions about which models to implement should, therefore, consider other factors like client costs and facility readiness.

Lastly, we clarify that “cost‐savings” in this model refers to reduced resource investment per ART client, not budget reductions. Freed resources can be reallocated within the healthcare system, but budgetary reductions should not be expected [26].

## 5 CONCLUSIONS

With 5 years remaining towards achieving the global UNAIDS 95‐95‐95 targets in 2030, many countries in sub‐Saharan Africa, such as Zambia, may benefit from restructuring their DSD model offerings for ART distribution [27]. The model presented here allows for rapid evaluation of different DSD allocations in terms of health outcomes, client costs and healthcare system costs, ensuring consideration of all sub‐populations. Given the scale of ART programmes and widespread DSD adoption in sub‐Saharan Africa, even small improvements in service delivery can have large impacts. Applying this model, based on existing data, may help drive such progress.

## COMPETING INTERESTS

The authors report no competing interests.

## AUTHORS’ CONTRIBUTIONS

NAL, SR, LJ and BEN conceptualized the study. NAL, SDM, CG, LJ and BEN developed the methodology. NAL, BP, AK, PH, HS and MM curated the data. NAL, SR, BP, AK, PH, HS, MM, PL‐M, AH, SP, LJ and BEN conducted the investigation. NAL, BP, PL‐M and LJ validated the data. NAL, LJ and BEN performed the formal analysis. SR, LJ and BEN supervised the study. SR, AH and SP acquired funding. LJ and BEN created the visualizations. NAL, LJ and BEN drafted the original manuscript. SR, SDM, CG, AK, PH, HS, MM, PL‐M, AH, SP, LJ and BEN reviewed and edited the final manuscript.

## FUNDING

Funding for the study was provided by the Bill & Melinda Gates Foundation through OPP1192640 to Boston University and INV‐037138 to the Wits Health Consortium. The funders played no role in the study design, data collection, analysis, interpretation or writing of the manuscript.

## Supporting information




**Text S1**: Cohort and outcomes
**Table S1**: Health outcomes—retention and viral suppression rates stratified by ART delivery model, sex, setting and age group
**Text S2**: Cost inputs
**Table S2**: Unit costs (provider costs)
**Table S3**: Average cost (2023 USD) to ART clients per year, stratified by ART delivery model
**Text S3**: DSD model allocation
**Table S4**: Distribution of clients among ART delivery models for the scenarios analysed
**Text S4**: Sensitivity analysis: cost inputs
**Table S5**: Confidence intervals for scenario on cost‐effectiveness frontier
**Table S6**: Cost‐effectiveness analysis of all scenarios
**Table S7**: Cost to clients per year for scenario on cost‐effectiveness frontier
**Text S5**: Health outcomes stratified by sub‐population
**Table S8**: Retention (a) and viral suppression (b) rates for scenarios on cost‐effectiveness frontier compared to the base case distribution by ART client sub‐category
**Text S6**: Sensitivity analysis: total provider costs and ICER per person suppressed
**Figure S1**: Sensitivity analysis of base case total provider costs
**Figure S2**: Sensitivity analysis of ICER per person suppressed on treatment for each of the scenarios on the cost‐effectiveness frontier

## Data Availability

The data that support the findings of this study are available from the Zambian Ministry of Health. Restrictions apply to the availability of these data, which were used under license for this study. Data are available from the author(s) with the permission of the Zambian Ministry of Health.
