# Supplementary material for: Cost and effectiveness of differentiated ART service delivery strategies in Zambia: a modelling analysis using routine data
Source: J Int AIDS Soc. 2025 Jun 28;28(7):e70003. doi: 10.1002/jia2.70003 (PMC12205209; doi:10.1002/jia2.70003)
Supplement: Supplementary file 1 — Text S1: Cohort and outcomes Table S1: Health outcomes—retention and viral suppression rates stratified by ART delivery model, sex, setting and age group Text S2: Cost inputs Table S2: Unit costs (provider costs) Table S3: Average cost (2023 USD) to ART clients per year, stratified by ART delivery model Text S3: DSD model allocation Table S4: Distribution of clients among ART delivery models for the scenarios analysed Text S4: Sensitivity analysis: cost inputs Table S5: Confidence intervals for scenario on cost‐effectiveness frontier Table S6: Cost‐effectiveness analysis of all scenarios Table S7: Cost to clients per year for scenario on cost‐effectiveness frontier Text S5: Health outcomes stratified by sub‐population Table S8: Retention (a) and viral suppression (b) rates for scenarios on cost‐effectiveness frontier compared to the base case distribution by ART client sub‐category Text S6: Sensitivity analysis: total provider costs and ICER per person suppressed Figure S1: Sensitivity analysis of base case total provider costs Figure S2: Sensitivity analysis of ICER per person suppressed on treatment for each of the scenarios on the cost‐effectiveness frontier [file JIA2-28-e70003-s001.docx]

**Supplementary Appendix**

**Cost and effectiveness of differentiated ART service delivery strategies in Zambia: a modelling analysis using routine data**

Nkgomeleng A Lekodeba^1^, Sydney Rosen^1,2^, Bevis Phiri^3^^, Sithabiso D Masuku^1,4^, Caroline Govathson^1,5^, Aniset Kamanga^3^, Prudence Haimbe^3^, Hilda Shakwelele^3^, Muya Mwansa^6^, Priscilla Lumano-Mulenga^6^, Amy Huber^1^, Sophie Pascoe^1^, Lise Jamieson^1,7*^, Brooke E Nichols^1,2,5*§^

1. Health Economics and Epidemiology Research Office, Faculty of Health Sciences, University of the Witwatersrand, Johannesburg, South Africa
2. Department of Global Health, Boston University School of Public Health, MA, United States
3. Clinton Health Access Initiative, Lusaka, Zambia
4. School of Health and Related Research, Health Economic and Decision Science (HEDS), The University of Sheffield, Sheffield, UK
5. Department of Global Health, Amsterdam Institute for Global Health and Development, Amsterdam UMC, University of Amsterdam, Amsterdam, Netherlands
6. Ministry of Health, Lusaka, Zambia
7. The South African Department of Science and Innovation/National Research Foundation Centre of Excellence in Epidemiological Modelling and Analysis (SACEMA), Stellenbosch University, Stellenbosch, South Africa

Contents

[Text S1: Cohort and outcomes 2](#_Toc199518418)

[Table S1. Health outcomes – retention and viral suppression rates stratified by ART delivery model, sex, setting and age group 2](#_Toc199518419)

[Text S2. Cost inputs 5](#_Toc199518420)

[Table S2. Unit costs (provider costs) 5](#_Toc199518421)

[Table S3. Average cost (2023 USD) to ART clients per year, stratified by ART delivery model 6](#_Toc199518422)

[Text S3. DSD model allocation 6](#_Toc199518423)

[Table S4. Distribution of clients among ART delivery models for the scenarios analysed 7](#_Toc199518424)

[Text S4: Sensitivity analysis: cost inputs 13](#_Toc199518425)

[Table S5. Confidence intervals for scenario on cost-effectiveness frontier 13](#_Toc199518426)

[Table S6. Cost-effectiveness analysis of all scenarios 14](#_Toc199518427)

[Table S7: Cost to clients per year for scenario on cost-effectiveness frontier 18](#_Toc199518428)

[Text S5: Health outcomes stratified by sub-population 18](#_Toc199518429)

[Table S8: Retention (a) and viral suppression (b) rates for scenarios on cost-effectiveness frontier compared to the base case distribution by ART client sub-category 19](#_Toc199518430)

[Text S6: Sensitivity analysis: total provider costs and ICER per person suppressed 19](#_Toc199518431)

[Figure S1. Sensitivity analysis of base case total provider costs 20](#_Toc199518432)

[Figure S2. Sensitivity analysis of ICER per person suppressed on treatment for each of the scenario on the cost-effectiveness frontier 20](#_Toc199518433)

[References 21](#_Toc199518434)

## **Text S1: Cohort and outcomes**

The distribution of ART delivery model which reflect the 2022 cohort by sex, settings, age and health outcomes (retention rates and suppression rates) were estimated from the Zambia electronic medical record – Smartcare database [1]. The proportion of ART clients who had a clinic visit between 11 and 24 months stratified by age group, sex, setting (urban/rural), and model of ART delivery. Retention in care for clients enrolled in DSD model was estimated after they became eligible and were enrolled in DSD models. Among those retained in care, the viral suppression rate was calculated. This included the proportion of ART clients who had viral load test results of <1,000 copies/ml at 12 months, stratified by age group, sex, setting (urban/rural), and model of ART delivery. The viral load at 12 months is measured between 11 and 24 months.

## **Table S1. Health outcomes – retention and viral suppression rates stratified by ART delivery model, sex, setting and age group**

| **ART delivery model** | **Sex** | **Setting** | **Age** | **Retention rates, % (95% CI)** | **Suppression rates, %**  **(95% CI)** |
| --- | --- | --- | --- | --- | --- |
| Scholar/adolescents model | Female | Rural | 15-19 | 94.4 (74.2; 99.0) | 91.7 (64.6; 98.5) |
| Fast track refills | Female | Rural | 15-19 | 76.5 (52.7; 98.4) | 95.7 (89.3; 99.4) |
| Conventional care not eligible for DSD | Female | Rural | 15-19 | 81.9 (78.5; 84.8) | 67.6 (62.0; 72.7) |
| Six-month dispensing (6MMD) | Female | Rural | 15-19 | 89.6 (86.8; 91.9) | 83.3 (77.9; 87.7) |
| Adherence groups | Female | Rural | 15-19 | 87.5 (64.0; 97.5) | 96.8 (94.5; 98.2) |
| Community ART distribution points | Female | Rural | 15-19 | 95.0 (76.4; 99.1) | 75.0 (46.8; 91.1) |
| Health posts | Female | Rural | 15-19 | 91.5 (85.1; 95.3) | 100.0 (89.3; 100.0) |
| Mobile ART distribution | Female | Rural | 15-19 | 94.9 (89.9; 97.5) | 92.0 (81.2; 96.8) |
| Conventional care (3MMD) | Female | Rural | 15-19 | 87.4 (85.3; 89.2) | 76.2 (72.6; 79.6) |
| Scholar/adolescents model | Male | Rural | 15-19 | 94.4 (74.2; 99.0) | 91.7 (64.6; 98.5) |
| Fast track refills | Male | Rural | 15-19 | 90.4 (82.1; 99.0) | 95.7 (93.0; 99.4) |
| Conventional care not eligible for DSD | Male | Rural | 15-19 | 74.6 (71.9; 77.1) | 82.7 (79.0; 85.9) |
| Six-month dispensing (6MMD) | Male | Rural | 15-19 | 88.5 (87.1; 89.8) | 94.2 (92.4; 95.6) |
| Adherence groups | Male | Rural | 15-19 | 97.2 (90.4; 99.2) | 96.8 (94.5; 98.2) |
| Community ART distribution points | Male | Rural | 15-19 | 88.9 (80.2; 94.0) | 83.3 (66.4; 92.7) |
| Health posts | Male | Rural | 15-19 | 91.5 (85.1; 95.3) | 100.0 (89.3; 100.0) |
| Mobile ART distribution | Male | Rural | 15-19 | 94.9 (89.9; 97.5) | 92.0 (81.2; 96.8) |
| Conventional care (3MMD) | Male | Rural | 15-19 | 84.0 (82.5; 85.4) | 87.1 (85.0; 88.9) |
| Scholar/adolescents model | Female | Urban | 15-19 | 82.4 (71.6; 89.6) | 96.7 (83.3; 99.4) |
| Fast track refills | Female | Urban | 15-19 | 92.3 (89.5; 97.8) | 95.7 (89.3; 97.4) |
| Conventional care not eligible for DSD | Female | Urban | 15-19 | 80.5 (79.9; 81.1) | 90.1 (89.5; 90.8) |
| Six-month dispensing (6MMD) | Female | Urban | 15-19 | 92.2 (90.0; 94.0) | 96.0 (93.0; 98.0) |
| Adherence groups | Female | Urban | 15-19 | 91.9 (90.4; 96.1) | 96.8 (94.5; 98.2) |
| Community ART distribution points | Female | Urban | 15-19 | 92.1 (90.6; 93.4) | 95.9 (94.1; 97.2) |
| Health posts | Female | Urban | 15-19 | 91.5 (85.1; 95.3) | 100.0 (89.3; 100.0) |
| Conventional care (3MMD) | Female | Urban | 15-19 | 97.1 (96.0; 99.0) | 92.8 (88.1; 96.6) |
| Scholar/adolescents model | Male | Urban | 15-19 | 94.9 (89.9; 97.5) | 92.0 (81.2; 96.8) |
| Fast track refills | Male | Urban | 15-19 | 87.5 (87.2; 87.8) | 92.4 (92.0; 92.7) |
| Conventional care not eligible for DSD | Male | Urban | 15-19 | 82.4 (71.6; 89.6) | 96.7 (83.3; 99.4) |
| Six-month dispensing (6MMD) | Male | Urban | 15-19 | 95.5 (91.9; 98.5) | 95.7 (93.0; 98.4) |
| Adherence groups | Male | Urban | 15-19 | 81.6 (80.4; 82.7) | 93.2 (92.1; 94.1) |
| Community ART distribution points | Male | Urban | 15-19 | 93.2 (91.0; 95.0) | 96.5 (93.9; 98.0) |
| Health posts | Male | Urban | 15-19 | 90.1 (87.2; 95.4) | 96.8 (94.5; 98.2) |
| Conventional care (3MMD) | Male | Urban | 15-19 | 91.8 (88.8; 94.1) | 99.5 (97.3; 99.9) |
| Scholar/adolescents model | Female | Rural | 20-24 | 91.5 (85.1; 95.3) | 100.0 (89.3; 100.0) |
| Fast track refills | Female | Rural | 20-24 | 85.7 (79.5; 98.0) | 92.8 (89.1; 96.6) |
| Conventional care not eligible for DSD | Female | Rural | 20-24 | 94.9 (89.9; 97.5) | 92.0 (81.2; 96.8) |
| Six-month dispensing (6MMD) | Female | Rural | 20-24 | 88.9 (88.4; 89.4) | 94.4 (93.9; 94.9) |
| Adherence groups | Female | Rural | 20-24 | 90.0 (59.6; 98.2) | 91.7 (64.6; 98.5) |
| Community ART distribution points | Female | Rural | 20-24 | 76.5 (62.7; 98.4) | 93.5 (78.9; 96.1) |
| Health posts | Female | Rural | 20-24 | 80.7 (77.0; 84.0) | 63.9 (58.2; 69.2) |
| Mobile ART distribution | Female | Rural | 20-24 | 92.5 (89.5; 94.7) | 83.2 (77.0; 88.1) |
| Conventional care (3MMD) | Female | Rural | 20-24 | 87.5 (64.0; 96.5) | 94.2 (90.0; 96.8) |
| Scholar/adolescents model | Male | Rural | 20-24 | 95.0 (76.4; 99.1) | 75.0 (46.8; 91.1) |
| Fast track refills | Male | Rural | 20-24 | 89.9 (81.3; 94.8) | 92.9 (68.5; 98.7) |
| Conventional care not eligible for DSD | Male | Rural | 20-24 | 95.7 (89.3; 98.3) | 87.5 (69.0; 95.7) |
| Six-month dispensing (6MMD) | Male | Rural | 20-24 | 90.4 (88.4; 92.1) | 73.1 (69.1; 76.8) |
| Adherence groups | Male | Rural | 20-24 | 90.0 (59.6; 98.2) | 91.7 (64.6; 98.5) |
| Community ART distribution points | Male | Rural | 20-24 | 90.4 (82.1; 98.0) | 93.5 (89.1; 96.1) |
| Health posts | Male | Rural | 20-24 | 75.9 (69.8; 81.1) | 63.0 (53.2; 71.8) |
| Mobile ART distribution | Male | Rural | 20-24 | 91.6 (88.6; 93.9) | 89.4 (83.3; 93.5) |
| Conventional care (3MMD) | Male | Rural | 20-24 | 97.2 (90.4; 99.2) | 94.2 (90.0; 96.8) |
| Scholar/adolescents model | Female | Urban | 20-24 | 88.9 (80.2; 94.0) | 83.3 (66.4; 92.7) |
| Fast track refills | Female | Urban | 20-24 | 89.9 (81.3; 94.8) | 92.9 (68.5; 98.7) |
| Conventional care not eligible for DSD | Female | Urban | 20-24 | 95.7 (89.3; 98.3) | 87.5 (69.0; 95.7) |
| Six-month dispensing (6MMD) | Female | Urban | 20-24 | 84.7 (81.7; 87.3) | 76.4 (71.3; 80.9) |
| Adherence groups | Female | Urban | 20-24 | 98.1 (89.9; 99.7) | 100.0 (85.1; 100.0) |
| Community ART distribution points | Female | Urban | 20-24 | 92.3 (89.0; 98.0) | 93.5 (78.9; 98.1) |
| Health posts | Female | Urban | 20-24 | 77.7 (76.8; 78.7) | 88.6 (87.5; 89.6) |
| Mobile ART distribution | Female | Urban | 20-24 | 91.7 (89.0; 95.6) | 95.3 (92.0; 98.0) |
| Conventional care (3MMD) | Female | Urban | 20-24 | 91.9 (90.4; 95.1) | 94.2 (90.0; 96.8) |
| Scholar/adolescents model | Male | Urban | 20-24 | 92.1 (90.6; 93.4) | 95.9 (94.1; 97.2) |
| Fast track refills | Male | Urban | 20-24 | 89.9 (81.3; 94.8) | 92.9 (68.5; 98.7) |
| Conventional care not eligible for DSD | Male | Urban | 20-24 | 92.6 (88.1; 99.0) | 92.8 (89.1; 96.6) |
| Six-month dispensing (6MMD) | Male | Urban | 20-24 | 95.7 (89.3; 98.3) | 87.5 (69.0; 95.7) |
| Adherence groups | Male | Urban | 20-24 | 86.0 (85.5; 86.4) | 91.6 (91.0; 92.1) |
| Community ART distribution points | Male | Urban | 20-24 | 98.1 (89.9; 99.7) | 100.0 (85.1; 100.0) |
| Health posts | Male | Urban | 20-24 | 95.5 (89.2; 98.5) | 93.5 (89.1; 96.1) |
| Conventional care (3MMD) | Male | Urban | 20-24 | 80.1 (78.8; 81.4) | 91.2 (89.9; 92.3) |
| Extended clinic hours | Female | Rural | 25-49 | 92.7 (90.1; 96.3) | 95.8 (92.0; 97.5) |
| Fast track refills | Female | Rural | 25-49 | 90.1 (87.2; 96.4) | 94.2 (90.0; 96.8) |
| Conventional care not eligible for DSD | Female | Rural | 25-49 | 91.8 (88.8; 94.1) | 99.5 (97.3; 99.9) |
| Six-month dispensing (6MMD) | Female | Rural | 25-49 | 89.9 (81.3; 94.8) | 92.9 (68.5; 98.7) |
| Adherence groups | Female | Rural | 25-49 | 90.5 (86.1; 98.3) | 92.8 (89.1; 96.6) |
| Community ART distribution points | Female | Rural | 25-49 | 95.7 (89.3; 98.3) | 87.5 (69.0; 95.7) |
| Health posts | Female | Rural | 25-49 | 88.2 (87.6; 88.7) | 93.3 (92.7; 93.9) |
| Home ART delivery | Female | Rural | 25-49 | 93.0 (81.4; 97.6) | 83.3 (66.4; 92.7) |
| Mobile ART distribution | Female | Rural | 25-49 | 88.2 (80.1; 98.3) | 96.8 (90.1; 97.3) |
| Conventional care (3MMD) | Female | Rural | 25-49 | 77.3 (75.2; 79.3) | 66.0 (62.4; 69.4) |
| Extended clinic hours | Male | Rural | 25-49 | 88.9 (86.8; 90.8) | 80.9 (76.6; 84.5) |
| Fast track refills | Male | Rural | 25-49 | 87.5 (64.0; 96.5) | 96.5 (93.2; 98.2) |
| Conventional care not eligible for DSD | Male | Rural | 25-49 | 95.0 (76.4; 99.1) | 75.0 (46.8; 91.1) |
| Six-month dispensing (6MMD) | Male | Rural | 25-49 | 89.0 (85.8; 91.5) | 93.7 (89.2; 96.3) |
| Adherence groups | Male | Rural | 25-49 | 86.0 (84.8; 87.1) | 74.4 (72.3; 76.4) |
| Community ART distribution points | Male | Rural | 25-49 | 93.0 (81.4; 97.6) | 83.3 (66.4; 92.7) |
| Health posts | Male | Rural | 25-49 | 90.3 (97.9; 97.3) | 96.8 (96.1; 97.3) |
| Home ART delivery | Male | Rural | 25-49 | 64.9 (63.1; 66.6) | 78.7 (76.2; 81.1) |
| Mobile ART distribution | Male | Rural | 25-49 | 87.9 (82.8; 96.9) | 93.1 (89.2; 97.3) |
| Conventional care (3MMD) | Male | Rural | 25-49 | 97.2 (90.4; 99.2) | 96.5 (93.2; 98.2) |
| Extended clinic hours | Female | Urban | 25-49 | 88.9 (80.2; 94.0) | 83.3 (66.4; 92.7) |
| Fast track refills | Female | Urban | 25-49 | 89.0 (85.8; 91.5) | 93.7 (89.2; 96.3) |
| Conventional care not eligible for DSD | Female | Urban | 25-49 | 80.0 (79.1; 81.0) | 86.1 (84.8; 87.4) |
| Six-month dispensing (6MMD) | Female | Urban | 25-49 | 82.4 (71.6; 89.6) | 96.7 (83.3; 99.4) |
| Adherence groups | Female | Urban | 25-49 | 92.2 (88.8; 99.0) | 96.8 (92.1; 98.3) |
| Community ART distribution points | Female | Urban | 25-49 | 76.1 (75.7; 76.5) | 89.7 (89.3; 90.1) |
| Health posts | Female | Urban | 25-49 | 90.4 (88.1; 96.2) | 95.4 (92.2; 97.7) |
| Home ART delivery | Female | Urban | 25-49 | 91.9 (87.0; 96.1) | 96.5 (93.2; 98.2) |
| Conventional care (3MMD) | Female | Urban | 25-49 | 92.1 (90.6; 93.4) | 95.9 (94.1; 97.2) |
| Extended clinic hours | Male | Urban | 25-49 | 89.0 (85.8; 91.5) | 93.7 (89.2; 96.3) |
| Fast track refills | Male | Urban | 25-49 | 94.7 (88.0; 98.2) | 97.3 (93.4; 99.1) |
| Conventional care not eligible for DSD | Male | Urban | 25-49 | 86.7 (86.6; 86.9) | 93.4 (93.2; 93.6) |
| Six-month dispensing (6MMD) | Male | Urban | 25-49 | 82.4 (71.6; 89.6) | 96.7 (83.3; 99.4) |
| Adherence groups | Male | Urban | 25-49 | 92.6 (89.1; 98.5) | 96.8 (96.1; 97.3) |
| Community ART distribution points | Male | Urban | 25-49 | 83.5 (82.8; 84.1) | 93.8 (93.1; 94.3) |
| Health posts | Male | Urban | 25-49 | 91.1 (88.1; 96.6) | 95.5 (92.9; 98.0) |
| Home ART delivery | Male | Urban | 25-49 | 90.1 (87.2; 96.4) | 96.5 (93.2; 98.2) |
| Conventional care (3MMD) | Male | Urban | 25-49 | 91.8 (88.8; 94.1) | 99.5 (97.3; 99.9) |
| Extended clinic hours | Female | Rural | 50+ | 89.0 (85.8; 91.5) | 93.7 (89.2; 96.3) |
| Fast track refills | Female | Rural | 50+ | 84.2 (83.2; 99.5) | 97.3 (92.4; 99.1) |
| Conventional care not eligible for DSD | Female | Rural | 50+ | 89.6 (89.3; 89.9) | 95.0 (94.7; 95.3) |
| Six-month dispensing (6MMD) | Female | Rural | 50+ | 96.7 (83.3; 99.4) | 83.3 (66.4; 92.7) |
| Adherence groups | Female | Rural | 50+ | 88.2 (78.0; 98.3) | 96.2 (90.3; 98.9) |
| Community ART distribution points | Female | Rural | 50+ | 79.2 (77.1; 81.1) | 63.3 (60.0; 66.4) |
| Health posts | Female | Rural | 50+ | 89.7 (87.3; 91.7) | 80.4 (75.8; 84.3) |
| Home ART delivery | Female | Rural | 50+ | 87.5 (64.0; 96.5) | 96.1 (91.2; 98.3) |
| Mobile ART distribution | Female | Rural | 50+ | 95.0 (76.4; 99.1) | 75.0 (46.8; 91.1) |
| Conventional care (3MMD) | Female | Rural | 50+ | 87.0 (81.9; 90.9) | 95.7 (89.3; 98.3) |
| Extended clinic hours | Male | Rural | 50+ | 87.8 (86.6; 88.9) | 74.7 (72.5; 76.8) |
| Fast track refills | Male | Rural | 50+ | 96.7 (83.3; 99.4) | 83.3 (66.4; 92.7) |
| Conventional care not eligible for DSD | Male | Rural | 50+ | 90.3 (79.0; 98.3) | 96.2 (92.9; 96.9) |
| Six-month dispensing (6MMD) | Male | Rural | 50+ | 72.6 (69.9; 75.2) | 69.3 (65.1; 73.2) |
| Adherence groups | Male | Rural | 50+ | 89.1 (87.0; 90.9) | 81.8 (77.3; 85.6) |
| Community ART distribution points | Male | Rural | 50+ | 97.2 (90.4; 99.2) | 96.1 (91.2; 98.3) |
| Health posts | Male | Rural | 50+ | 88.9 (80.2; 94.0) | 83.3 (66.4; 92.7) |
| Home ART delivery | Male | Rural | 50+ | 87.0 (81.9; 90.9) | 95.7 (89.3; 98.3) |
| Mobile ART distribution | Male | Rural | 50+ | 81.8 (80.2; 83.3) | 74.1 (71.4; 76.6) |
| Conventional care (3MMD) | Male | Rural | 50+ | 98.1 (89.9; 99.7) | 100.0 (85.1; 100.0) |
| Extended clinic hours | Female | Urban | 50+ | 92.2 (78.9; 99.0) | 96.2 (90.3; 99.3) |
| Fast track refills | Female | Urban | 50+ | 74.8 (74.2; 75.3) | 88.1 (87.5; 88.7) |
| Conventional care not eligible for DSD | Female | Urban | 50+ | 89.5 (79.2; 96.9) | 94.5 (92.1; 97.9) |
| Six-month dispensing (6MMD) | Female | Urban | 50+ | 91.9 (90.4; 97.1) | 96.1 (91.2; 98.3) |
| Adherence groups | Female | Urban | 50+ | 92.1 (90.6; 93.4) | 95.9 (94.1; 97.2) |
| Community ART distribution points | Female | Urban | 50+ | 87.0 (81.9; 90.9) | 95.7 (89.3; 98.3) |
| Health posts | Female | Urban | 50+ | 90.7 (86.5; 94.8) | 97.3 (95.4; 99.1) |
| Home ART delivery | Female | Urban | 50+ | 84.5 (84.3; 84.8) | 92.0 (91.7; 92.3) |
| Conventional care (3MMD) | Female | Urban | 50+ | 98.1 (89.9; 99.7) | 100.0 (85.1; 100.0) |
| Extended clinic hours | Male | Urban | 50+ | 92.6 (89.1; 96.5) | 96.2 (90.3; 96.9) |
| Fast track refills | Male | Urban | 50+ | 81.8 (81.0; 82.5) | 91.7 (91.0; 92.4) |
| Conventional care not eligible for DSD | Male | Urban | 50+ | 90.4 (84.9; 96.0) | 94.8 (90.1; 98.4) |
| Six-month dispensing (6MMD) | Male | Urban | 50+ | 90.1 (87.2; 98.4) | 96.1 (91.2; 98.3) |
| Adherence groups | Male | Urban | 50+ | 91.8 (88.8; 94.1) | 99.5 (97.3; 99.9) |
| Community ART distribution points | Male | Urban | 50+ | 87.0 (81.9; 90.9) | 95.7 (89.3; 98.3) |
| Health posts | Male | Urban | 50+ | 91.7 (86.5; 98.5) | 97.3 (94.2; 99.1) |
| Home ART delivery | Male | Urban | 50+ | 88.5 (88.2; 88.8) | 93.5 (93.1; 93.9) |
| Conventional care (3MMD) | Male | Urban | 50+ | 94.4 (74.2; 99.0) | 91.7 (64.6; 98.5) |

## **Text S2. Cost inputs**

Provider costs using ingredients-based approach and costs to clients were estimated using previously published data (Table S2 and Table S3, respectively) [2,3]. Provider costs per ART client per year included costs of staff time (facility visits and DSD interactions), medication costs, and laboratory testing costs (Table S2) [3]. Costs to clients are based on self-reported resource utilization collected during the SENTINEL survey [2] . Clients in each ART delivery models were asked to report the number of interactions (visits) with the healthcare system, the time spent accessing care at each study site and if whether they incurred any transport costs [2,4]. Cost to clients included opportunity costs for time spent seeking care, estimated as time spent accessing care multiplied by Zambia’s minimum daily wage ($1.99), and transport costs incurred for accessing ART services [2], taking into consideration the proportion of clients incurring transport costs in each ART delivery model (Table S3). Unit costs for the provider and costs to ART clients per year were updated to 2023 prices [2,3,5] and then converted from Zambian Kwacha (ZMW) to United States Dollars (USD) using the average 2023 exchange rate of 20.23 ZMW per = 1 USD [5].

## **Table S2. Unit costs (provider costs)**

| **Cost Items** | **Mean unit cost**  **(2023 USD)** | **Description** |
| --- | --- | --- |
| **Laboratory (cost per test)** | | Assumed one test per person per year [6]. |
| Viral load | $27.44 |  |
| CD4 count | $5.38 |  |
| **HIV treatment** |  | Assumed first-line regimen (Tenofovir/Lamivudine/  Dolutegravir) for all clients [6]. |
| ART (per month) | $5.25 |  |
| **Interaction costs** | **Mean unit cost (range)** |  |
| **Facility visit (cost per visit)** | | Unit cost data were collected from previously published literature in Zambia and updated with 2023 public sector prices and staff costs [3]. Facility visit and DSD interaction costs include staff time, equipment, consumables, overheads, and training costs [3]. |
| Clinical follow-up | $2.71 (1.90; 3.53) |  |
| Short clinic visit | $1.93 (1.29; 2.56) |  |
| Pharmacy visit | $0.59 (0.41; 0.77) |  |
| **DSD interaction (cost per interaction)** | |  |
| Scholar/adolescent model | $1.11 (0.74; 1.49) |  |
| Fast-track refills | $1.93 (1.29; 2.56) |  |
| Adherence groups^†^ | $1.73 (1.08; 2.38) |  |
| Community ART distribution points‡ | $2.35 (1.43; 3.28) |  |
| Health post§ | $2.71 (1.90; 3.53) |  |
| Home ART delivery | $11.13 (5.74; 16.53) |  |
| Mobile ART distribution | $8.81 (7.36; 10.26) |  |
| † assumed similar resources use as Community Adherence groups and urban adherence groups  ‡ assumed similar resources use as urban adherence groups  § assumed similar resources use as clinical follow-up | | |

**Table S3. Average cost (2023 USD) to ART clients per year, stratified by ART delivery model**

| Model of care | N | Opportunity cost/cost to clients/year† (mean, SD) | | Transport costs/cost to clients/year (mean, 95% CI) | |
| --- | --- | --- | --- | --- | --- |
|  |  | **Time spent (hours)** | **Mean cost**  **(95% CI)** | **% client incurring any transport costs**** | **Travel costs/client incurring any transport costs** |
| Conventional care not eligible for DSD | 66 | 27.9 | $6.94 (5.60; 8.28) | 51.5% | $3.19 (1.98; 4.40) |
| Conventional care (3MMD) | 66 | 20.28 | $5.01 (4.33; 5.70) | 40.9% | $3.01 (0.89; 5.14) |
| Six-month dispensing | 118 | 12.16 | $3.01 (2.50; 3.51) | 36.4% | $2.80 (1.56; 4.04) |
| Scholar/adolescent model | 41 | 17.55 | $4.34 (3.19; 5.48) | 26.8% | $1.66 (0.64; 2.68) |
| Adherence groups | 23 | 26.06 | $6.44 (4.45; 8.42) | 34.8% | $2.09 (0.14; 4.31) |
| Community ART distribution points‡ | 37 | 21.10 | $5.21 (3.94; 6.49) | 51.4% | $1.71 (1,04; 2.38) |
| Extended clinic hours | 15 | 9.52 | $2.35 (1.94; 2.76) | 46.7% | $1.74 (0.56; 2.91) |
| Fast track refills | 31 | 15.96 | $3.94 (2.94; 4.95) | 45.2% | $1.32 (0.86; 1.77) |
| Mobile ART | 9 | 16.23 | $4.01 (2.10; 5.92) | 0.0% | $0.00 (0.00; 0.00) |
| Home ART delivery | 25 | 10.82 | $2.67 (2.19; 3.16) | 40.0% | $2.98 (1.16; 4.81) |
| Health post§ | 37 | 21.10 | $5.21 (3.94; 6.49) | 51.4% | $1.71 (1,04; 2.38) |
| Cost to clients data was collected and updated from previously collected data [3]  *Includes both clinical consult visits and ART medication pick up visits at the facility.  **Remainder likely walked, incurred no cash costs.  †Calculated opportunity cost based on the cost of time spent travelling and time at clinic visits or out-of-facility events. The minimum wage for Zambia of $1.98/day (adjusted to an hourly minimum wage using 8 working hours/day) as used to assign a monetary value to the time spent.  ‡Community adherence access points are equivalent to external medication pickup points.  § assumed similar resources use as Community ART distribution points | | | | | |

## **Text S3. DSD model allocation**

We modelled two different sets of scenarios: 1) DSD models for the full population (excluding DSD models that are targeted to specific population subgroups); and 2) stratified scenarios which included age-specific DSD models for relevant subgroups and other DSD models for everyone else. For scenarios with two or more DSD models, we assumed equal distribution of the number of eligible ART clients allocated to each model.

Each scenario reflected a different allocation of eligible clients to the models included in the scenario. In most scenarios, 95% of ART clients were allocated to differentiated models (non-conventional care), with the rest remaining in conventional care. For example, in a fast-track refills-only scenario, all eligible clients were enrolled in fast-track refills and the remainder were in conventional care. In a population specific scenario such as enrolling everyone eligible for DSD in a combination of scholar/adolescent model and extended clinic hours, eligible clients aged ≤24 years were enrolled in scholar/adolescent model and those aged ≥25 years enrolled in extended clinic hours. The details of mix of ART delivery models and client distribution by scenario are described in Table S4.

## **Table S4. Distribution of clients among ART delivery models for the scenarios analysed**

| Scenario number | Scenario name | Descriptions/DSD mix and distribution of ART clients |
| --- | --- | --- |
| Base case | 2022 ART distribution | The current ART program in Zambia which includes 10 ART delivery models plus conventional care (3MMD and frequent refills) distribution as indicated in 2022 SmartCare database.   - 80.2% DSD coverage, 14.1% in conventional care eligible for DSD but not enrolled, and 5.3% in conventional care not eligible for DSD. |
| *Scenario 1-4: The scenarios tailored to clients based on model population-specific enrolment with different DSD coverage (e.g. scholar/adolescent model is tailored for clients aged ≤24 years).* | | |
| 1 | Scholar/adolescent model-only | Enrolling all eligible clients aged ≤24 years in scholar/adolescent model   - 6.8% DSD coverage for clients aged ≤24 years, 90.1% conventional care (3MMD), and 3.1% remain in conventional care not eligible |
| 2 | MAD-only | Enrolling all eligible clients in rural settings in mobile ART delivery   - 29.3% DSD coverage of all clients in rural settings, 70.7% in conventional care (3MMD), and 3.1% remain in conventional care not eligible |
| 3 | HAD-only | Enrolling all eligible clients aged ≥25 years in Home ART delivery   - 87.9% DSD coverage for clients aged ≥25+, 9.0% conventional care (3MMD), and 3.1% remains in conventional care not eligible for DSD |
| 4 | ECH-only | Enrolling all eligible clients aged ≥25 years in extended clinic hours   - 87.9% DSD coverage for clients aged ≥25+, 9.0% conventional care (3MMD), and 3.1% remains in conventional care not eligible for DSD |
| *Scenario 5-9: Scenarios for all eligible clients, utilising DSD models with no age/setting specific restrictions, enrolling all clients in one DSD model at a time. Clients distribution: 94.7% DSD coverage and 5.3% conventional care not eligible for DSD.* | | |
| 5 | FTRs-only | Enrolling all eligible clients in fast-track refills |
| 6 | 6MMD-only | Enrolling all eligible clients in 6MMD |
| 7 | CADP-only | Enrolling all eligible clients in community ART distribution points |
| 8 | HP-only | Enrolling all eligible clients in health post |
| 9 | AGs-only | Enrolling all eligible clients in adherence groups |
| *Scenario 10-19: Scenario 5-9: Scenarios for all eligible clients, utilising DSD models with no age/setting specific restrictions, enrolling all clients in two DSD models at a time -* *paired all possible combinations. Clients distribution: 94.7% DSD coverage and 5.3% conventional care not eligible for DSD.* | | |
| 10 | FTRs & 6MMD | Equal distribution of all eligible clients between fast track refills and 6MMD |
| 11 | FTRs & AGs | Equal distribution of all eligible clients between fast track refills and adherence groups |
| 12 | FTRs & CADP | Equal distribution of all eligible clients between fast track refills and community ART distribution points |
| 13 | FTRs & HP | Equal distribution of all eligible clients between fast track refills and health posts |
| 14 | 6MMD & AGs | Equal distribution of all eligible clients between 6MMD and adherence groups |
| 15 | 6MMD & CADP | Equal distribution of all eligible clients between 6MMD and community ART distribution points |
| 16 | 6MMD & HP | Equal distribution of all eligible clients between 6MMD and health posts |
| 17 | AGs & CADP | Equal distribution of all eligible clients between adherence groups and community ART distribution points |
| 18 | AGs & HP | Equal distribution of all eligible clients between adherence groups and health posts |
| 19 | CADP & HP | Equal distribution of all eligible clients between community ART distribution points and health posts |
| *Scenario 20-29: Scenarios for all eligible clients, utilising DSD models with no age/setting specific restrictions, enrolling all clients in three DSD models at a time - paired all possible combinations. Clients distribution: 94.7% DSD coverage and 5.3% conventional care not eligible for DSD.* | | |
| 20 | FTRs, 6MMD & AGs | Equal distribution of all eligible clients between fast-track refills, 6MMD and adherence groups |
| 21 | FTRs, 6MMD & CADP | Equal distribution of all eligible clients between fast-track refills, 6MMD and community ART distribution points |
| 22 | FTRs, 6MMD & HP | Equal distribution of all eligible clients between fast-track refills, 6MMD and health posts |
| 23 | FTRs, AGs & CADP | Equal distribution of all eligible clients between fast-track refills, adherence groups and community ART distribution points |
| 24 | FTR, AGs & HP | Equal distribution of all eligible clients between fast-track refills, adherence groups and health posts |
| 25 | FTRs, CADP & HP | Equal distribution of all eligible clients between fast-track refills, community ART distribution points and health post |
| 26 | 6MMD, AGs & CADP | Equal distribution of all eligible clients between 6MMD, adherence groups and community ART distribution points |
| 27 | 6MMD, AGs & HP | Equal distribution of all eligible clients between 6MMD, adherence groups and health posts |
| 28 | 6MMD, CADP & HP | Equal distribution of all eligible clients between 6MMD, community ART distribution points and health posts |
| 29 | AGs, CADP & HP | Equal distribution of all eligible clients between Adherence groups, community ART distribution points and health posts |
| *Scenario 30-35: Scenarios for all eligible clients, utilising DSD models with no age/setting specific restrictions, enrolling all clients in four DSD models at a time - paired all possible combinations. Clients distribution: 94.7% DSD coverage and 5.3% conventional care not eligible for DSD.* | | |
| 30 | FTRs, MMD6, AGs & CADP | Equal distribution of all eligible clients between fast track refills, 6MMD, adherence groups and community ART distribution points |
| 31 | FTRs, MMD6, AGs & HP | Equal distribution of all eligible clients between fast track refills, 6MMD, adherence groups and health posts |
| 32 | FTRs, MMD6, CADP & HP | Equal distribution of all eligible clients between fast track refills, 6MMD, community ART distribution points and health posts |
| 33 | FTRs, AGs, CADP & HP | Equal distribution of all eligible clients between fast track refills, adherence groups, community ART distribution points and health posts |
| 34 | MMD6, AGs, CADP & HP | Equal distribution of all eligible clients between 6MMD, adherence groups, community ART distribution points and health posts |
|  | ***Scenario 35****: Scenario for all eligible clients, utilising DSD models with no age/setting specific restrictions, enrolling all clients in five DSD models at a time. Clients distribution: 94.7% DSD coverage and 5.3% conventional care not eligible for DSD.* | |
| 35 | FTRs, MMD6, AGs, CADP & HP | Equal distribution of all eligible clients between fast track refills, 6MMD, adherence groups, community ART distribution points and health posts |
| *Scenario 36-42: Age-specific scenarios - paired all possible combinations. Clients distribution: 94.7% DSD coverage (7.1% scholar/adolescent model & 92.9% in each of the DSD model) and 5.3% conventional care not eligible for DSD.* | | |
| 36 | Scholar/adolescent model & FTRs | Enrolling all eligible clients aged ≤24 years in scholar model and clients aged ≥25 years in fast tract refills |
| 37 | Scholar/adolescent model & 6MMD | Enrolling all eligible clients aged ≤24 years in scholar model and clients aged ≥25 years in 6MMD |
| 38 | Scholar/adolescent model & AGs | Enrolling all eligible clients aged ≤24 years in scholar model and clients aged ≥25 years in adherence groups |
| 39 | Scholar/adolescent model & CADP | Enrolling all eligible clients aged ≤24 years in scholar model and all clients aged ≥25 years in community ART distribution points |
| 40 | Scholar/adolescent model & HP | Enrolling all eligible clients aged ≤24 years in scholar model and clients aged ≥25 years in health posts |
| 41 | Scholar/adolescent model & ECH | Enrolling all eligible clients aged ≤24 years in scholar model and clients aged ≥25 years in extended clinic hours |
| 42 | Scholar/adolescent model & HAD | Enrolling all eligible clients aged ≤24 years in scholar model and eligible clients aged ≥25 years in Home ART delivery |
| *Scenario 43-47: scenarios enrolling all eligible clients in a mix of DSD model with age restrictions and models without age restrictions, distributing clients equally across- paired all possible combinations. Clients distribution: 94.7% DSD coverage (3.6% scholar/adolescent model & 96.4% in each DSD model) and 5.3% conventional care not eligible for DSD.* | | |
| 43 | Scholar/adolescent model & fast track refills | Equal distribution of clients aged ≤24 years between scholar/adolescent model and fast track refills and enrolling eligible clients aged ≥25 years in fast track refills |
| 44 | Scholar/adolescent model & 6MMD | Equal distribution of clients aged ≤24 years between scholar/adolescent model and fast track refills and enrolling eligible clients aged ≥25 years in 6MMD |
| 45 | Scholar/adolescent model & AGs | Equal distribution of clients aged ≤24 years between scholar/adolescent model and fast track refills and enrolling eligible clients aged ≥25 years in adherence groups |
| 46 | Scholar/adolescent model & CADP | Equal distribution of clients aged ≤24 years between scholar/adolescent model and fast track refills and enrolling eligible clients aged ≥25 years in community ART distribution points |
| 47 | Scholar/adolescent model & HP | Equal distribution of clients aged ≤24 years between scholar/adolescent model and fast track refills and enrolling eligible clients aged ≥25 years in health posts |
| *Scenario 48-57:* *Age-specific scenarios - paired all possible combinations. Clients distribution: 94.7% DSD coverage (7.1% in each DSD models & 92.9% in either extended clinic hours and Home ART delivery) and 5.3% conventional care not eligible for DSD.* | | |
| 48 | FTRs & ECH | Enrolling all eligible clients aged ≤24 years in fast track refills and clients aged ≥25 years in extended clinic hours |
| 49 | AGs & ECH | Enrolling all eligible clients aged ≤24 years in adherence groups and clients aged ≥25 years in extended clinic hours |
| 50 | CADP & ECH | Enrolling all eligible clients aged ≤24 years in community ART distribution points and clients aged ≥25 years in extended clinic hours |
| 51 | HP & ECH | Enrolling all eligible clients aged ≤24 years in health posts and clients aged ≥25 years in extended clinic hours |
| 52 | 6MMD & ECH | Enrolling all eligible clients aged ≤24 years in 6MMD and clients aged ≥25 years in extended clinic hours |
| 53 | FTRs & HAD | Enrolling all eligible clients aged ≤24 years in fast track refills and clients aged ≥25 years in home ART delivery |
| 54 | CADP & HAD | Enrolling all eligible clients aged ≤24 years in community ART distribution points and clients aged ≥25 years in home ART delivery |
| 55 | AGs & HAD | Enrolling all eligible clients aged ≤24 years in adherence groups and clients aged ≥25 years in home ART delivery |
| 56 | HP & HAD | Enrolling all eligible clients aged ≤24 years in health posts and clients aged ≥25 years in home ART delivery |
| 57 | 6MMD & HAD | Enrolling all eligible clients aged ≤24 years in 6MMD and clients aged ≥25 years in home ART delivery |
| *Scenario 58-87: Age-specific scenarios - paired all possible combinations. Clients distribution: 94.7% DSD coverage (7.1% of clients aged ≤24 in distributed equally in each of the two models & 92.9% of clients in either home ART delivery or extended clinic hours) and 5.3% conventional care not eligible for DSD.* | | |
| 58 | Scholar/adolescent model, FTRs & ECH | Equal distribution of client’s eligible clients aged ≤24 years between scholar/adolescent model and fast track refills and clients aged ≥25 years in extended clinic hours |
| 59 | Scholar/adolescent model, AGs & ECH | Equal distribution of clients aged ≤24 years between scholar/adolescent model and adherence groups and clients aged ≥25 years in extended clinic hours |
| 60 | Scholar/adolescent model, CADP & ECH | Equal distribution of clients aged ≤24 years between scholar/adolescent model and community ART distribution points and clients aged ≥25 years in extended clinic hours |
| 61 | Scholar/adolescent model, 6MMD & ECH | Equal distribution of clients aged ≤24 years between scholar/adolescent model and 6MMD and clients aged ≥25 years in extended clinic hours |
| 62 | Scholar/adolescent model, HP & ECH | Equal distribution of clients aged ≤24 years between scholar/adolescent model and health posts and clients aged ≥25 years in home ART delivery |
| 63 | FTRs, AGs & ECH | Equal distribution of clients aged ≤24 years between fast track refills and adherence groups and clients aged ≥25 years in extended clinic hours |
| 64 | FTRs, CADP & ECH | Equal distribution of clients aged ≤24 years between fast track refills and community adherence groups and clients aged ≥25 years in extended clinic hours |
| 65 | FTRs, 6MMD & ECH | Equal distribution of clients aged ≤24 years between fast track refills and 6MMD and clients aged ≥25 years in extended clinic hours |
| 66 | AGs, CADP & ECH | Equal distribution of clients aged ≤24 years between adherence groups and community adherence groups and clients aged ≥25 years in extended clinic hours |
| 67 | AGS, 6MMD & ECH | Equal distribution of clients aged ≤24 years between adherence groups and 6MMD and clients aged ≥25 years in extended clinic hours |
| 68 | CADP, 6MMD & ECH | Equal distribution of clients aged ≤24 years between community ART distribution points and 6MMD and clients aged ≥25 years in extended clinic hours |
| 69 | Scholar/adolescent model, HP & ECH | Equal distribution of clients aged ≤24 years between scholar/adolescent model and health posts and clients aged ≥25 years in extended clinic hours |
| 70 | FTRs, HP & ECH | Equal distribution of clients aged ≤24 years between scholar/adolescent model and health posts and clients aged ≥25 years in extended clinic hours |
| 71 | AGs, HP & ECH | Equal distribution of clients aged ≤24 years between adherence groups and health posts and clients aged ≥25 years in extended clinic hours |
| 72 | CADP, HP & ECH | Equal distribution of clients aged ≤24 years between community adherence groups and health posts and clients aged ≥25 years in extended clinic hours |
| 73 | 6MMD, HP & ECH | Equal distribution of clients aged ≤24 years between 6MMD and health posts and clients aged ≥25 years in extended clinic hours |
| 73 | Scholar/adolescent model, FTRs & HAD | Equal distribution of clients aged ≤24 years between scholar/adolescent model and fast track refills and clients aged ≥25 years in home ART delivery |
| 74 | Scholar/adolescent model, AGS & HAD | Equal distribution of clients aged ≤24 years between scholar/adolescent model and adherence groups and clients aged ≥25 years in home ART delivery |
| 75 | Scholar/adolescent model, CADP & HAD | Equal distribution of clients aged ≤24 years between scholar/adolescent model and community ART distribution points and clients aged ≥25 years in home ART delivery |
| 76 | Scholar/adolescent model, 6MMD & HAD | Equal distribution of clients aged ≤24 years between scholar/adolescent model and 6MMD and clients aged ≥25 years in home ART delivery |
| 77 | FTRs, AGs & HAD | Equal distribution of clients aged ≤24 years between fast track refills and adherence groups and clients aged ≥25 years in home ART delivery |
| 78 | FTRs, CADP & HAD | Equal distribution of clients aged ≤24 years between fast track refills and community ART distribution points and clients aged ≥25 years in home ART delivery |
| 79 | FTRs, 6MMD & HAD | Equal distribution of clients aged ≤24 years between fast track refills and c and clients aged ≥25 years in home ART delivery |
| 80 | AGs, CADP & HAD | Equal distribution of clients aged ≤24 years between adherence groups and community ART distribution points and clients aged ≥25 years in home ART delivery |
| 81 | AGs, 6MMD & HAD | Equal distribution of clients aged ≤24 years between adherence groups and 6MMD and clients aged ≥25 years in home ART delivery |
| 82 | CADP, 6MMD & HAD | Equal distribution of clients aged ≤24 years between community ART distribution points and 6MMD and clients aged ≥25 years in home ART delivery |
| 83 | Scholar/adolescent model, HP & HAD | Equal distribution of clients aged ≤24 years between scholar/adolescent model and health posts and clients aged ≥25 years in home ART delivery |
| 84 | FTRs, HP & HAD | Equal distribution of clients aged ≤24 years between fast track refills and health posts and clients aged ≥25 years in home ART delivery |
| 85 | AGs, HP& HAD | Equal distribution of clients aged ≤24 years between fast track refills and health posts and clients aged ≥25 years in home ART delivery |
| 86 | CADP, HP & HAD | Equal distribution of clients aged ≤24 years between community ART distribution points and health posts and clients aged ≥25 years in home ART delivery |
| 87 | 6MMD, HP & HAD | Equal distribution of clients aged ≤24 years between 6MMD and health posts and clients aged ≥25 years in home ART delivery |
| *Scenario 88-108: Age-specific scenarios - paired all possible combinations. Clients distribution: 94.7% DSD coverage (7.1% scholar/adolescent model & 92.9% each of the two models) and 5.3% conventional care not eligible for DSD.* | | |
| 88 | Scholar/adolescent model, FTRs & AGs | Clients aged ≤24 years in scholar/adolescent and equal distribution of clients aged ≥25 years in fast track refills and adherence groups. |
| 89 | Scholar/adolescent model, FTRs & CADP | Clients aged ≤24 years in scholar/adolescent and equal distribution of clients aged ≥25 years in fast track refills and community ART distribution points. |
| 90 | Scholar/adolescent model, FTRs & 6MMD | Clients aged ≤24 years in scholar/adolescent and equal distribution of clients aged ≥25 years in fast track refills and 6MMD. |
| 91 | Scholar/adolescent model, FTRs & HP | Clients aged ≤24 years in scholar/adolescent and equal distribution of clients aged ≥25 years in fast track refills and health posts. |
| 92 | Scholar/adolescent model, FTRs & HAD | Clients aged ≤24 years in scholar/adolescent and equal distribution of clients aged ≥25 years in fast track refills and home ART delivery. |
| 93 | Scholar/adolescent model, AGs & CADP | Clients aged ≤24 years in scholar/adolescent and equal distribution of clients aged ≥25 years in adherence groups and community ART distribution points. |
| 94 | Scholar/adolescent model, AGs & 6MMD | Clients aged ≤24 years in scholar/adolescent and equal distribution of clients aged ≥25 years in adherence groups and 6MMD. |
| 95 | Scholar/adolescent model, AGs & HP | Clients aged ≤24 years in scholar/adolescent and equal distribution of clients aged ≥25 years in adherence groups and health posts. |
| 96 | Scholar/adolescent model, AGs & HAD | Clients aged ≤24 years in scholar/adolescent and equal distribution of clients aged ≥25 years in community ART distribution points and home ART delivery. |
| 97 | Scholar/adolescent model, CADP & 6MMD | Clients aged ≤24 years in scholar/adolescent and equal distribution of clients aged ≥25 years in community ART distribution points and 6MMD. |
| 98 | Scholar/adolescent model, CADP & HP | Clients aged ≤24 years in scholar/adolescent and equal distribution of clients aged ≥25 years in community ART distribution points and health posts. |
| 99 | Scholar/adolescent model, CADP & HAD | Clients aged ≤24 years in scholar/adolescent and equal distribution of clients aged ≥25 years in community ART distribution points and home ART delivery. |
| 100 | Scholar/adolescent model, 6MMD & HP | Clients aged ≤24 years in scholar/adolescent and equal distribution of clients aged ≥25 years in 6MMD and health posts. |
| 101 | Scholar/adolescent model, 6MMD & HAD | Clients aged ≤24 years in scholar/adolescent and equal distribution of clients aged ≥25 years in 6MMD and home ART delivery. |
| 102 | Scholar/adolescent model, HP & HAD | Clients aged ≤24 years in scholar/adolescent and equal distribution of clients aged ≥25 years in health posts and home ART delivery. |
| 103 | Scholar/adolescent model, FTRs & ECH | Clients aged ≤24 years in scholar/adolescent and equal distribution of clients aged ≥25 years in fast track refills and extended clinic hours. |
| 104 | Scholar/adolescent model, AGs & ECH | Clients aged ≤24 years in scholar/adolescent and equal distribution of clients aged ≥25 years in adherence groups and extended clinic hours. |
| 105 | Scholar/adolescent model, CADP & ECH | Clients aged ≤24 years in scholar/adolescent and equal distribution of clients aged ≥25 years in community ART distribution points and extended clinic hours. |
| 106 | Scholar/adolescent model, 6MMD & ECH | Clients aged ≤24 years in scholar/adolescent and equal distribution of clients aged ≥25 years in 6MMD and extended clinic hours. |
| 107 | Scholar/adolescent model, HP & ECH | Clients aged ≤24 years in scholar/adolescent and equal distribution of clients aged ≥25 years in health posts and extended clinic hours. |
| 108 | Scholar/adolescent model, HAD & ECH | Clients aged ≤24 years in scholar/adolescent and equal distribution of clients aged ≥25 years in home ART delivery and extended clinic hours. |
| *Scenario 109-118: Settings-specific scenarios - paired all possible combinations. Clients distribution: 94.7% DSD coverage and 5.3% conventional care not eligible for DSD (30.9% mobile ART delivery & 69.1% in each of DSD models)* | | |
| 109 | MAD & FTRs | Equal distribution of clients in rural area between mobile ART delivery and fast track refills and clients in urban areas only in fast track refills. |
| 110 | MAD & MMD6 | Equal distribution of clients in rural area between mobile ART delivery and 6MMD and clients in urban areas only in 6MMD. |
| 111 | MAD & AGs | Equal distribution of clients in rural area between mobile ART delivery and adherence groups and clients in urban areas only in adherence groups. |
| 112 | MAD & CADP | Equal distribution of clients in rural area between mobile ART delivery and community ART distribution points and clients in urban areas only in community ART distribution points. |
| 113 | MAD & HP | Equal distribution of clients in rural area between mobile ART delivery and health posts and clients in urban areas only in health posts. |
| 114 | MAD & FTRs | Enrolling all eligible clients in rural areas in mobile ART delivery and clients in urban areas in fast track refills. |
| 115 | MAD & 6MMD | Enrolling all eligible clients in rural areas in mobile ART delivery and clients in urban areas in 6MMD. |
| 116 | MAD & AGs | Enrolling all eligible clients in rural areas in mobile ART delivery and clients in urban areas in adherence groups. |
| 117 | MAD & CADP | Enrolling all eligible clients in rural areas in mobile ART delivery and clients in urban areas in community ART distribution points. |
| 118 | MAD & HP | Enrolling all eligible clients in rural areas in mobile ART delivery and clients in urban areas in health posts. |
| *Scenario 119-125: Settings and age specific scenarios - paired all possible combinations. Clients distribution: 94.7% DSD coverage and 5.3% conventional care not eligible for DSD (4.8% scholar/adolescent model, 64.3% each of the DSD model & 30.9% mobile ART delivery).* | | |
| 119 | Scholar/adolescent model, MAD & FTRs | Enrolling all eligible clients aged ≤24 in urban areas in scholar/adolescent model and clients all clients in rural areas in mobile ART delivery and clients aged ≥25 years in urban settings in fast track refills. |
| 120 | Scholar/adolescent model, MAD & 6MMD | Enrolling all eligible clients aged ≤24 in urban areas in scholar/adolescent model and clients all clients in rural areas in mobile ART delivery and clients aged ≥25 years in urban settings in 6MMD. |
| 121 | Scholar/adolescent model, MAD & AGs | Enrolling all eligible clients aged ≤24 in urban areas in scholar/adolescent model and clients all clients in rural areas in mobile ART delivery and clients aged ≥25 years in urban settings in adherence groups. |
| 122 | Scholar/adolescent model, MAD & CADP | Enrolling all eligible clients aged ≤24 in urban areas in scholar/adolescent model and clients all clients in rural areas in mobile ART delivery and clients aged ≥25 years in urban settings in community ART distribution points. |
| 123 | Scholar/adolescent model, MAD & HP | Enrolling all eligible clients aged ≤24 in urban areas in scholar/adolescent model and clients all clients in rural areas in mobile ART delivery and clients aged ≥25 years in urban settings in health posts. |
| 124 | Scholar/adolescent model, MAD & ECH | Enrolling all eligible clients aged ≤24 in urban areas in scholar/adolescent model and clients all clients in rural areas in mobile ART delivery and clients aged ≥25 years in urban settings in extended clinic hours. |
| 125 | Scholar/adolescent model, MAD & HAD | Enrolling all eligible clients aged ≤24 in urban areas in scholar/adolescent model and clients all clients in rural areas in mobile ART delivery and clients aged ≥25 years in urban settings in Home ART delivery. |

## **Text S4: Sensitivity analysis: cost inputs**

For the total provider costs under the base case scenario and ICER per person suppressed on treatment for the scenarios on the cost-effectiveness frontier, respectively. We varied model input cost parameters using estimated lower and upper bounds from literature which included costs of ART, laboratory test costs, DSD interaction and facility visits costs (Table S2), modelling input assumptions – client distributions and number of visits or DSD interactions per year and health outcome (retention and suppression rates).

**Table S5. Confidence intervals for scenario on cost-effectiveness frontier**

| **Scenario** | **Total suppressed (95% CI)** |
| --- | --- |
| Base case | 770,086 (717,521; 823,735) |
| 6MMD | 782,545 (724,788; 846,146) |
| 6MMD & AGs | 791,712 (733,632; 851,998) |
| AGs | 800,878 (742,477; 866,063) |
| FTRs & AGs | 802,755 (730,983; 865,189) |
| FTRs | 804,632 (736,924; 874,277) |
| AGs & HAD | 807,356 (745,765; 871,801) |

## **Table S6. Cost-effectiveness analysis of all scenarios**

| **Scenario** | **Scenario name** | **Total number retained** | **Total number suppressed** | **Total Provider costs** | **Total cost to Clients** | **ICER** |
| --- | --- | --- | --- | --- | --- | --- |
| 6 | 6MMD-only | 827,415 | 782,545 | $83,095,136 | $3,455,290 | Cost-saving (compared to base case) |
| 44 | Scholar/adolescent model & 6MMD | 828,965 | 783,204 | $83,521,537 | $3,675,394 | Weakly dominated |
| 37 | Scholar/adolescent model & 6MMD | 830,515 | 783,863 | $83,947,939 | $3,895,498 | Weakly dominated |
| Base case | Current distribution | 817,948 | 770,086 | $84,332,234 | $4,145,400 | Strongly dominated |
| 16 | 6MMD & HP | 820,325 | 777,551 | $85,309,798 | $4,325,380 | Strongly dominated |
| 14 | 6MMD & AGs | 831,498 | 791,712 | $85,336,549 | $6,756,112 | $245 |
| 94 | Scholar/adolescent model, AGs & 6MMD | 832,904 | 789,246 | $85,885,529 | $6,948,089 | Strongly dominated |
| 100 | Scholar/adolescent model, 6MMD & HP | 823,264 | 776,761 | $85,937,093 | $4,700,546 | Strongly dominated |
| 27 | 6MMD, AGs & HP | 825,410 | 785,327 | $86,065,853 | $6,235,898 | Strongly dominated |
| 106 | Scholar/adolescent model, 6MMD & ECH | 819,734 | 783,130 | $86,192,538 | $3,553,640 | Strongly dominated |
| 15 | 6MMD & CADP | 832,870 | 790,662 | $86,222,602 | $4,404,699 | Strongly dominated |
| 10 | FTRs & 6MMD | 834,142 | 793,588 | $86,241,860 | $4,426,956 | Weakly dominated |
| 28 | 6MMD, CADP & HP | 826,325 | 784,627 | $86,656,555 | $4,668,290 | Strongly dominated |
| 22 | FTRs, 6MMD & HP | 827,173 | 786,578 | $86,669,394 | $4,683,127 | Strongly dominated |
| 26 | 6MMD, AGs & CADP | 833,774 | 794,068 | $86,674,388 | $6,288,777 | Weakly dominated |
| 20 | FTRs, 6MMD & AGs | 834,622 | 796,018 | $86,687,227 | $6,303,615 | Weakly dominated |
| 110 | MAD & MMD6 | 831,633 | 780,024 | $86,822,887 | $3,473,242 | Strongly dominated |
| 97 | Scholar/adolescent model, CADP & 6MMD | 835,379 | 793,669 | $86,834,300 | $4,777,146 | Strongly dominated |
| 34 | MMD6, AGs, CADP & HP | 828,639 | 788,690 | $86,886,906 | $6,015,451 | Strongly dominated |
| 31 | FTRs, MMD6, AGs & HP | 829,275 | 790,153 | $86,896,535 | $6,026,579 | Strongly dominated |
| 90 | Scholar/adolescent model, FTRs & 6MMD | 837,332 | 792,891 | $86,933,743 | $4,802,730 | Strongly dominated |
| 21 | FTRs, 6MMD & CADP | 835,536 | 795,319 | $87,277,929 | $4,736,006 | Strongly dominated |
| 1 | Scholar/adolescent model-only | 798,346 | 736,820 | $87,280,581 | $5,581,838 | Strongly dominated |
| 32 | FTRs, MMD6, CADP & HP | 829,961 | 789,628 | $87,339,562 | $4,850,873 | Strongly dominated |
| 30 | FTRs, MMD6, AGs & CADP | 835,548 | 796,709 | $87,352,937 | $6,066,238 | Weakly dominated |
| 35 | FTRs, MMD6, AGs, CADP & HP | 831,085 | 791,878 | $87,387,242 | $5,892,085 | Strongly dominated |
| 8 | HP-only | 813,234 | 772,556 | $87,524,461 | $5,195,471 | Strongly dominated |
| 18 | AGs & HP | 824,408 | 786,717 | $87,551,211 | $7,626,202 | Strongly dominated |
| 9 | AGs-only | 835,581 | 800,878 | $87,577,961 | $10,056,934 | $245 |
| 52 | 6MMD & ECH | 805,851 | 781,080 | $87,584,333 | $2,771,574 | Strongly dominated |
| 45 | Scholar/adolescent model & AGs | 835,437 | 797,753 | $87,700,540 | $10,028,807 | Strongly dominated |
| 47 | Scholar/adolescent model & HP | 814,623 | 771,108 | $87,725,354 | $5,350,533 | Strongly dominated |
| 4 | ECH-only | 802,451 | 774,257 | $87,728,203 | $2,891,995 | Strongly dominated |
| 64 | FTRs, CADP & ECH | 805,762 | 783,094 | $87,745,254 | $2,836,008 | Strongly dominated |
| 72 | CADP, HP & ECH | 806,013 | 783,186 | $87,809,842 | $2,836,616 | Strongly dominated |
| 38 | Scholar/adolescent model & AGs | 835,293 | 794,629 | $87,823,118 | $10,000,680 | Strongly dominated |
| 67 | AGS, 6MMD & ECH | 806,443 | 779,390 | $87,825,439 | $2,839,334 | Strongly dominated |
| 95 | Scholar/adolescent model, AGs & HP | 825,652 | 782,144 | $87,874,682 | $7,753,137 | Strongly dominated |
| 66 | AGs, CADP & ECH | 807,546 | 784,863 | $87,888,156 | $3,019,805 | Strongly dominated |
| 48 | FTRs & ECH | 805,673 | 785,109 | $87,906,174 | $2,900,441 | Strongly dominated |
| 40 | Scholar/adolescent model & HP | 816,012 | 769,660 | $87,926,246 | $5,505,595 | Strongly dominated |
| 69 | Scholar/adolescent model, HP & ECH | 805,924 | 785,201 | $87,970,762 | $2,901,050 | Strongly dominated |
| 63 | FTRs, AGs & ECH | 806,354 | 781,405 | $87,986,359 | $2,903,768 | Strongly dominated |
| 61 | Scholar/adolescent model, 6MMD & ECH | 807,401 | 781,738 | $88,010,735 | $2,991,678 | Strongly dominated |
| 51 | HP & ECH | 806,174 | 785,293 | $88,035,351 | $2,901,658 | Strongly dominated |
| 62 | Scholar/adolescent model, HP & ECH | 807,457 | 786,878 | $88,049,076 | $3,084,238 | Strongly dominated |
| 71 | AGs, HP & ECH | 806,604 | 781,497 | $88,050,947 | $2,904,376 | Strongly dominated |
| 50 | CADP & ECH | 807,034 | 777,701 | $88,066,544 | $2,907,095 | Strongly dominated |
| 70 | FTRs, HP & ECH | 807,707 | 786,970 | $88,113,665 | $3,084,847 | Strongly dominated |
| 65 | FTRs, 6MMD & ECH | 808,137 | 783,174 | $88,129,262 | $3,087,565 | Strongly dominated |
| 104 | Scholar/adolescent model, AGs & ECH | 822,122 | 788,513 | $88,130,127 | $6,606,231 | Strongly dominated |
| 29 | AGs, CADP & HP | 829,047 | 790,738 | $88,150,830 | $6,868,838 | Strongly dominated |
| 24 | FTR, AGs & HP | 829,895 | 792,689 | $88,163,669 | $6,883,675 | Strongly dominated |
| 58 | Scholar/adolescent model, FTRs & ECH | 807,313 | 783,753 | $88,171,655 | $3,056,111 | Strongly dominated |
| 107 | Scholar/adolescent model, HP & ECH | 812,482 | 776,029 | $88,181,691 | $4,358,688 | Strongly dominated |
| 49 | AGs & ECH | 809,240 | 788,646 | $88,191,979 | $3,268,035 | Strongly dominated |
| 68 | CADP, 6MMD & ECH | 807,563 | 783,845 | $88,236,244 | $3,056,720 | Strongly dominated |
| 60 | Scholar/adolescent model, CADP & ECH | 807,993 | 780,049 | $88,251,840 | $3,059,438 | Strongly dominated |
| 59 | Scholar/adolescent model, AGs & ECH | 809,096 | 785,522 | $88,314,558 | $3,239,909 | Strongly dominated |
| 41 | Scholar/adolescent model & ECH | 808,952 | 782,397 | $88,437,136 | $3,211,782 | Strongly dominated |
| 19 | CADP & HP | 825,779 | 785,668 | $88,437,264 | $5,274,789 | Strongly dominated |
| 13 | FTRs & HP | 827,052 | 788,594 | $88,456,522 | $5,297,046 | Strongly dominated |
| 33 | FTRs, AGs, CADP & HP | 832,002 | 794,211 | $88,460,268 | $6,501,284 | Strongly dominated |
| 17 | AGs & CADP | 836,953 | 799,829 | $88,464,015 | $7,705,521 | Strongly dominated |
| 11 | FTRs & AGs | 838,225 | 802,755 | $88,483,273 | $7,727,778 | $482 |
| 25 | FTRs, CADP & HP | 830,809 | 791,989 | $88,754,371 | $5,316,067 | Strongly dominated |
| 93 | Scholar/adolescent model, AGs & CADP | 837,767 | 799,052 | $88,771,889 | $7,829,738 | Strongly dominated |
| 23 | FTRs, AGs & CADP | 838,258 | 801,430 | $88,772,204 | $6,936,554 | Strongly dominated |
| 98 | Scholar/adolescent model, CADP & HP | 828,127 | 786,568 | $88,823,453 | $5,582,195 | Strongly dominated |
| 88 | Scholar/adolescent model, FTRs & AGs | 839,720 | 798,274 | $88,871,333 | $7,855,321 | Strongly dominated |
| 91 | Scholar/adolescent model, FTRs & HP | 830,080 | 785,790 | $88,922,896 | $5,607,778 | Strongly dominated |
| 105 | Scholar/adolescent model, CADP & ECH | 824,597 | 792,936 | $89,078,898 | $4,435,288 | Strongly dominated |
| 103 | Scholar/adolescent model, FTRs & ECH | 826,550 | 792,158 | $89,178,341 | $4,460,872 | Strongly dominated |
| 7 | CADP-only | 838,325 | 798,779 | $89,350,068 | $5,354,108 | Strongly dominated |
| 12 | FTRs & CADP | 839,597 | 801,705 | $89,369,326 | $5,376,365 | Strongly dominated |
| 5 | FTRs-only | 840,869 | 804,632 | $89,388,584 | $5,398,622 | $482 |
| 46 | Scholar/adolescent model & CADP | 839,283 | 801,127 | $89,535,364 | $5,506,451 | Strongly dominated |
| 43 | Scholar/adolescent model & fast track refills | 842,508 | 803,276 | $89,654,065 | $5,554,292 | Strongly dominated |
| 39 | Scholar/adolescent model & CADP | 840,242 | 803,475 | $89,720,660 | $5,658,795 | Strongly dominated |
| 89 | Scholar/adolescent model, FTRs & CADP | 842,195 | 802,698 | $89,820,103 | $5,684,379 | Strongly dominated |
| 36 | Scholar/adolescent model & FTRs | 844,148 | 801,920 | $89,919,547 | $5,709,962 | Strongly dominated |
| 113 | MAD & HP | 818,973 | 769,095 | $90,481,332 | $4,935,811 | Strongly dominated |
| 115 | MAD & 6MMD | 835,851 | 777,502 | $90,550,639 | $3,491,194 | Strongly dominated |
| 111 | MAD & AGs | 840,344 | 798,362 | $90,792,565 | $9,060,995 | Strongly dominated |
| 120 | Scholar/adolescent model, MAD & 6MMD | 838,126 | 777,829 | $91,140,416 | $3,785,923 | Strongly dominated |
| 109 | MAD & FTRs | 844,409 | 802,197 | $92,280,254 | $5,120,643 | Strongly dominated |
| 112 | MAD & CADP | 842,674 | 796,220 | $92,291,788 | $5,085,661 | Strongly dominated |
| 2 | MAD-only | 813,319 | 744,752 | $93,139,757 | $4,743,071 | Strongly dominated |
| 118 | MAD & HP | 824,713 | 765,634 | $93,438,203 | $4,676,150 | Strongly dominated |
| 123 | Scholar/adolescent model, MAD & HP | 827,013 | 763,581 | $93,754,323 | $4,886,029 | Strongly dominated |
| 116 | MAD & AGs | 845,106 | 795,845 | $94,007,169 | $8,065,055 | Strongly dominated |
| 121 | Scholar/adolescent model, MAD & AGs | 845,007 | 790,668 | $94,180,566 | $8,028,637 | Strongly dominated |
| 124 | Scholar/adolescent model, MAD & ECH | 826,467 | 781,074 | $94,573,568 | $3,325,743 | Strongly dominated |
| 114 | MAD & FTRs | 847,949 | 799,763 | $95,171,925 | $4,842,665 | Strongly dominated |
| 117 | MAD & CADP | 847,024 | 793,661 | $95,233,508 | $4,817,215 | Strongly dominated |
| 119 | Scholar/adolescent model, MAD & FTRs | 849,742 | 796,324 | $95,484,499 | $5,048,018 | Strongly dominated |
| 122 | Scholar/adolescent model, MAD & CADP | 848,365 | 795,783 | $95,487,386 | $5,021,027 | Strongly dominated |
| 101 | Scholar/adolescent model, 6MMD & HAD | 837,418 | 792,485 | $96,883,656 | $4,632,777 | Strongly dominated |
| 96 | Scholar/adolescent model, AGs & HAD | 839,807 | 797,868 | $98,821,245 | $7,685,368 | Strongly dominated |
| 102 | Scholar/adolescent model, HP & HAD | 830,166 | 785,383 | $98,872,809 | $5,437,825 | Strongly dominated |
| 108 | Scholar/adolescent model, HAD & ECH | 826,636 | 791,752 | $99,128,254 | $4,290,919 | Strongly dominated |
| 99 | Scholar/adolescent model, CADP & HAD | 842,281 | 802,291 | $99,770,016 | $5,514,426 | Strongly dominated |
| 92 | Scholar/adolescent model, FTRs & HAD | 844,234 | 801,513 | $99,869,459 | $5,540,009 | Strongly dominated |
| 54 | CADP & HAD | 841,220 | 799,789 | $108,966,569 | $4,929,848 | Strongly dominated |
| 125 | Scholar/adolescent model, MAD & HAD | 848,478 | 798,981 | $109,046,436 | $4,805,072 | Strongly dominated |
| 3 | HAD-only | 837,820 | 792,967 | $109,110,439 | $5,050,269 | Strongly dominated |
| 79 | FTRs, CADP & HAD | 841,131 | 801,804 | $109,127,489 | $4,994,282 | Strongly dominated |
| 87 | CADP, HP & HAD | 841,382 | 801,896 | $109,192,078 | $4,994,891 | Strongly dominated |
| 82 | AGs, 6MMD & HAD | 841,811 | 798,100 | $109,207,675 | $4,997,609 | Strongly dominated |
| 81 | AGs, CADP & HAD | 842,915 | 803,573 | $109,270,392 | $5,178,079 | Strongly dominated |
| 53 | FTRs & HAD | 841,042 | 803,818 | $109,288,410 | $5,058,716 | Strongly dominated |
| 84 | Scholar/adolescent model, HP & HAD | 841,293 | 803,910 | $109,352,998 | $5,059,324 | Strongly dominated |
| 78 | FTRs, AGs & HAD | 841,722 | 800,114 | $109,368,595 | $5,062,042 | Strongly dominated |
| 76 | Scholar/adolescent model, CADP & HAD | 842,770 | 800,448 | $109,392,971 | $5,149,952 | Strongly dominated |
| 57 | 6MMD & HAD | 841,543 | 804,002 | $109,417,587 | $5,059,933 | Strongly dominated |
| 77 | Scholar/adolescent model, 6MMD & HAD | 842,826 | 805,587 | $109,431,312 | $5,242,513 | Weakly dominated |
| 86 | AGs, HP& HAD | 841,973 | 800,206 | $109,433,183 | $5,062,651 | Strongly dominated |
| 56 | HP & HAD | 842,403 | 796,410 | $109,448,780 | $5,065,369 | Strongly dominated |
| 85 | FTRs, HP & HAD | 843,076 | 805,679 | $109,495,901 | $5,243,121 | Strongly dominated |
| 80 | FTRs, 6MMD & HAD | 843,506 | 801,883 | $109,511,497 | $5,245,840 | Strongly dominated |
| 73 | 6MMD, HP & ECH | 842,681 | 802,462 | $109,553,891 | $5,214,386 | Strongly dominated |
| 55 | AGs & HAD | 844,609 | 807,356 | $109,574,215 | $5,426,310 | $7,409 |
| 83 | CADP, 6MMD & HAD | 842,932 | 802,554 | $109,618,479 | $5,214,995 | Strongly dominated |
| 75 | Scholar/adolescent model, AGS & HAD | 843,362 | 798,758 | $109,634,076 | $5,217,713 | Strongly dominated |
| 74 | Scholar/adolescent model, FTRs & HAD | 844,465 | 804,231 | $109,696,793 | $5,398,183 | Strongly dominated |
| 42 | Scholar/adolescent model & HAD | 844,321 | 801,106 | $109,819,372 | $5,370,056 | Strongly dominated |
| 6MMD, six months dispensing; MAD, Mobile ART delivery, HAD, Home ART delivery, ECH, extended clinic hours, FTRs, fast track refills; CADP, community ART distribution points, HP, health posts, AGs, adherence groups | | | | | | |

## **Table S7: Cost to clients per year for scenario on cost-effectiveness frontier**

| Scenarios | Cost to clients (95% CI) | Change in costs compared to base case (95% CI) |
| --- | --- | --- |
| Base case | $3,969,727  ($3,071,187; $4,951,395) | N/A |
| 6MMD | $3,492,944  ($2,740,270; $4,336,701) | -12.0%  (-10.8; -12.4) |
| 6MMD & AGs | $4,769,043  ($3,362,777; $6,345,200) | 20.1%  (9.5; 28.1) |
| AGs | $6,045,142  ($3,985,283; $8,353,698) | 52.3%  (29.8; 68.7) |
| FTRs & AGs | $4,977,633  ($3,416,366; $6,734,050) | 25.4%  (11.6; 36.0) |
| FTRs | $3,910,125  ($2,847,449; $5,114,402) | -1.5%  (-6.5; 3.3) |
| AGs & HAD | $3,736,007  ($2,592,495; $4,977,952) | -5.9%  (-14.6; 0.5) |

**Text S5: Health outcomes stratified by sub-population**


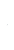

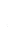
The relative impact of each scenario varied by age, sex, and setting (Table S7). For clients aged 25-49 years, who constituted over 70% of the ART population included in the analysis, all scenarios on the cost-effectiveness frontier resulted in increased retention compared to the base case. Retention was lower in female clients aged 15-19 years and male clients 15-24 in rural settings for all scenarios on the cost-effectiveness frontier except for 6MMD-only, which improved retention across all sub-populations. A scenario utilizing AGs-only had a low retention rate among clients aged ≥50+ in both urban and rural settings. Viral suppression increased for most subpopulations in most scenarios on the cost-effectiveness frontier, though 6MMD was slightly less successful for older clients and rural clients showed slightly lower suppression rates in several of the scenarios. 6MMD combined with adherence groups was associated with better viral suppression for the largest proportion of the subpopulations assessed except for male clients aged ≥50+ in rural settings.

**Table S8: Retention (a) and viral suppression (b) rates for scenarios on cost-effectiveness frontier compared to the base case distribution by ART client sub-category** †*

| **Sex/age/setting subgroup** | **Base case distribution** | **6MMD** | **6MMD & AGs** | **AGs** | **FTRs & AGs** | **FTRs** | **AGs & HAD** |
| --- | --- | --- | --- | --- | --- | --- | --- |
| **(a) Retention** | | | | | | | |
| Total | 89.1% | 90.2% | 90.6% | 91.0% | 91.3% | 91.6% | 92.0% |
| Male, 15-19, Rural | 90.6% | 91.7% | 89.5% | 87.2% | 82.3% | 77.3% | 87.2% |
| Male, 20-24, Rural | 88.6% | 90.2% | 92.7% | 95.1% | 92.1% | 89.1% | 95.1% |
| Male, 25-49, Rural | 89.8% | 91.1% | 91.2% | 91.3% | 91.5% | 91.8% | 92.1% |
| Male, 50+, Rural | 91.7% | 92.5% | 91.2% | 89.9% | 92.5% | 95.2% | 90.3% |
| Male, 15-19, Urban | 87.8% | 88.8% | 87.8% | 86.8% | 87.1% | 87.4% | 86.8% |
| Male, 20-24, Urban | 85.7% | 87.5% | 91.0% | 94.5% | 91.5% | 88.5% | 94.5% |
| Male, 25-49, Urban | 87.8% | 88.9% | 90.0% | 91.1% | 91.2% | 91.4% | 89.9% |
| Male, 50+, Urban | 90.1% | 90.3% | 90.1% | 89.9% | 91.2% | 92.4% | 91.5% |
| Female, 15-19, Rural | 88.0% | 89.0% | 88.0% | 87.1% | 82.3% | 77.5% | 87.1% |
| Female, 20-24, Rural | 85.9% | 87.5% | 91.4% | 95.4% | 92.3% | 89.2% | 95.4% |
| Female, 25-49, Rural | 90.7% | 91.8% | 91.7% | 91.5% | 91.7% | 91.9% | 96.5% |
| Female, 50+, Rural | 92.4% | 93.1% | 91.5% | 90.0% | 92.6% | 95.3% | 85.7% |
| Female, 15-19, Urban | 86.3% | 87.7% | 87.1% | 86.5% | 86.8% | 87.1% | 86.5% |
| Female, 20-24, Urban | 82.6% | 85.5% | 89.5% | 93.4% | 90.5% | 87.6% | 93.4% |
| Female, 25-49, Urban | 88.8% | 89.8% | 90.5% | 91.2% | 91.4% | 91.6% | 93.9% |
| Female, 50+, Urban | 90.8% | 91.0% | 90.5% | 90.0% | 91.3% | 92.5% | 84.2% |
| **(b) Viral suppression** | | | | | | | |
| Total | 94.1% | 94.6% | 95.2% | 95.8% | 95.8% | 95.7% | 95.6% |
| Male, 15-19, Rural | 80.3% | 81.9% | 86.7% | 91.8% | 91.3% | 90.7% | 91.8% |
| Male, 20-24, Rural | 84.6% | 87.3% | 89.6% | 91.7% | 91.3% | 90.9% | 91.7% |
| Male, 25-49, Rural | 94.3% | 95.1% | 94.6% | 94.0% | 93.7% | 93.3% | 92.6% |
| Male, 50+, Rural | 95.3% | 95.7% | 95.0% | 94.2% | 93.8% | 93.4% | 92.8% |
| Male, 15-19, Urban | 78.5% | 78.9% | 85.8% | 92.8% | 92.9% | 92.9% | 92.8% |
| Male, 20-24, Urban | 80.5% | 80.6% | 87.2% | 93.4% | 93.3% | 93.3% | 93.4% |
| Male, 25-49, Urban | 93.9% | 94.2% | 95.0% | 95.8% | 95.8% | 95.9% | 96.9% |
| Male, 50+, Urban | 94.8% | 94.7% | 95.4% | 96.0% | 96.1% | 96.1% | 97.2% |
| Female, 15-19, Rural | 80.2% | 82.0% | 87.8% | 93.7% | 93.1% | 92.4% | 93.7% |
| Female, 20-24, Rural | 91.5% | 93.4% | 94.6% | 95.8% | 95.3% | 94.7% | 95.8% |
| Female, 25-49, Rural | 95.2% | 95.8% | 96.2% | 96.6% | 96.0% | 95.5% | 92.7% |
| Female, 50+, Rural | 96.3% | 96.5% | 96.6% | 96.8% | 96.2% | 95.7% | 92.8% |
| Female, 15-19, Urban | 78.4% | 79.3% | 86.0% | 92.7% | 92.9% | 93.0% | 92.7% |
| Female, 20-24, Urban | 90.0% | 91.8% | 93.4% | 94.9% | 95.0% | 95.1% | 94.9% |
| Female, 25-49, Urban | 94.9% | 95.2% | 95.7% | 96.2% | 96.4% | 96.5% | 97.0% |
| Female, 50+, Urban | 95.7% | 95.5% | 96.0% | 96.5% | 96.6% | 96.7% | 97.3% |
| †based on mid-point value of health outcomes (retention and viral suppression rate)  *Cells shaded green are greater than base case; cells shaded red are less than base case. | | | | | | | |

**Text S6: Sensitivity analysis: total provider costs and ICER per person suppressed**

The main cost drivers for the base case were retention rate, cost of ART, VL tests, and clinical follow-up, while DSD interaction costs for HAD and AGs had minimal impact (Fig S1, S2). For 6MMD-only, combined 6MMD and AGs, AGs-only, and 6MMD+AGs+FTRs scenarios, laboratory test and ART costs were the most influential, affecting potential cost savings or increased costs per person suppressed (Fig 5a-d). For FTRs-only, the cost of fast-track visits was the key driver (Fig 5e). In the most expensive scenario (AGs+HAD), DSD interaction costs were the primary driver of ICER, with no potential for cost-saving (Fig 5f).

## **Figure S1. Sensitivity analysis of base case total provider costs**

(the dashed line represents the total provider costs of $84,332,234 under the base case scenario)


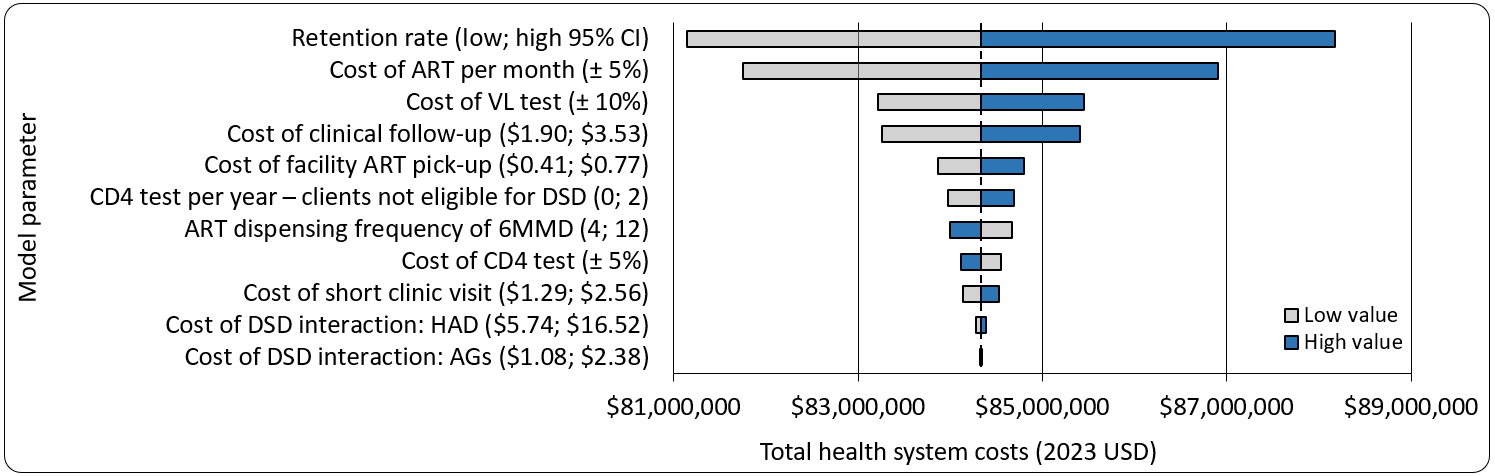


**Figure S2. Sensitivity analysis of ICER per person suppressed on treatment for each of the scenario on the cost-effectiveness frontier**

[The dashed line represents the uncertainty impact on the ICER under 6MMD-only (a), a combination of 6MMD and AGs (b), AGs-only (c), a combination of FTRs and AGs (d), FTRs-only (e), and a combination of AGs and HAD (f)]


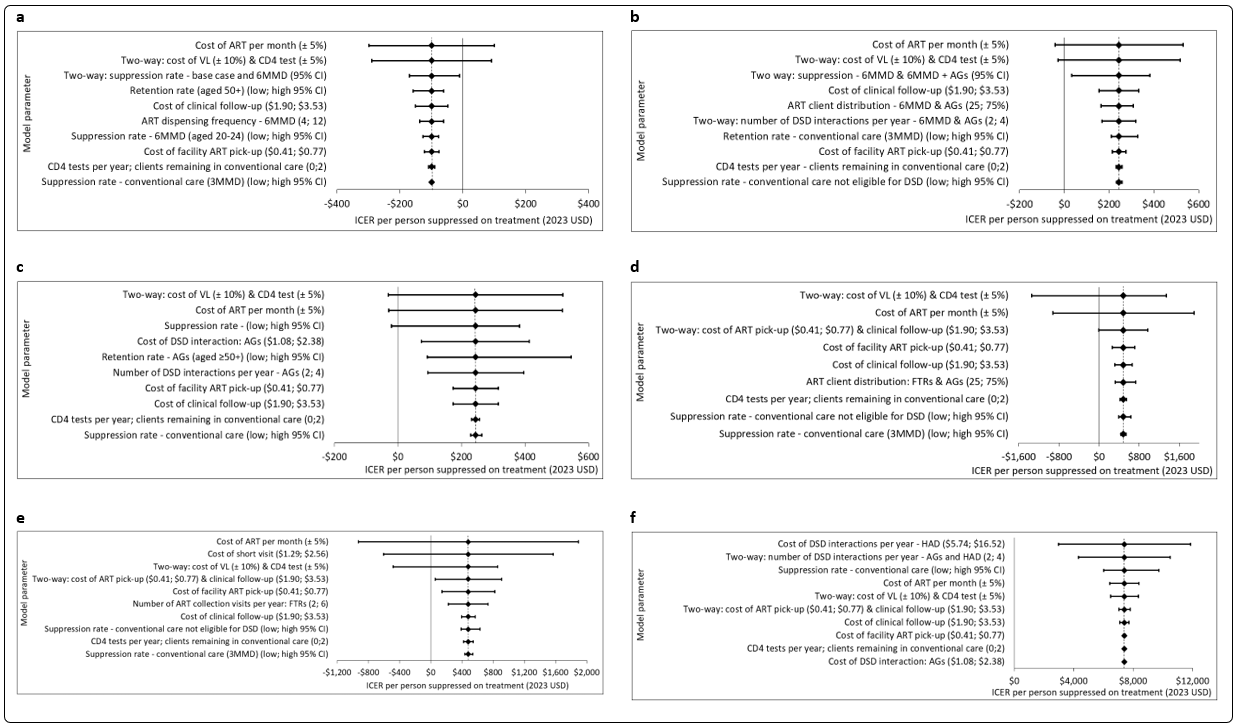


## **References**

1. Kaumba PC. Factors affecting the implementation of the SmartCare EHR system in Zambia. Social Sciences and Humanities Open. Elsevier Ltd; 2023. doi:10.1016/j.ssaho.2023.100399

2. Hendrickson C, Phiri B, Lekodeba N, Mokhele I, Huber A, Ntjikelane V, et al. Do differentiated models of care for HIV treatment result in lower costs for recipients of care in Zambia?. Abstract PESUE23. Montreal, Canada: 24th International AIDS Conference; 2022.

3. Nichols BE, Cele R, Jamieson L, Long LC, Siwale Z, Banda P, et al. Community-based delivery of HIV treatment in Zambia: costs and outcomes. AIDS. 2021;35: 299–306. doi:10.1097/QAD.0000000000002737

4. Pascoe S, Huber A, Mokhele I, Lekodeba N, Ntjikelane V, Sande L, et al. The SENTINEL study of differentiated service delivery models for HIV treatment in Malawi, South Africa, and Zambia: research protocol for a prospective cohort study. BMC Health Serv Res. 2023;23: 891. doi:10.1186/s12913-023-09813-w

5. Bank of Zambia. Historical Series of Exchange Rates. [cited 2 Feb 2023]. Available: https://www.boz.zm/average-exchange-rates.htm

6. Campbell J, Estill J, Panos Z, Harwell J, Chimhundu C, Shakwelele H, et al. Use of generic ritonavir-boosted darunavir and dolutegravir for second line antiretroviral therapy is cost-effective in Zambia : a 10-year modelling analysis Methods : ACORA model Methods : scenarios and inputs Results : Health Outcomes Discussion and Lim. 2022.
